# State of the Art and Prospects in Metal-Organic Framework-Derived Microwave Absorption Materials

**DOI:** 10.1007/s40820-022-00808-6

**Published:** 2022-02-26

**Authors:** Shuning Ren, Haojie Yu, Li Wang, Zhikun Huang, Tengfei Lin, Yudi Huang, Jian Yang, Yichuan Hong, Jinyi Liu

**Affiliations:** State Key Laboratory of Chemical Engineering, College of Chemical and Biological Engineering, Zhejiang University, Hangzhou, 310027 People’s Republic of China

**Keywords:** Microwave absorption materials, Metal–organic frameworks, Preparation methods, Mechanisms of microwave absorption

## Abstract

Microwave has been widely used in many fields, including communication, medical treatment and military industry; however, the corresponding generated radiations have been novel hazardous sources of pollution threating human’s daily life. Therefore, designing high-performance microwave absorption materials (MAMs) has become an indispensable requirement. Recently, metal–organic frameworks (MOFs) have been considered as one of the most ideal precursor candidates of MAMs because of their tunable structure, high porosity and large specific surface area. Usually, MOF-derived MAMs exhibit excellent electrical conductivity, good magnetism and sufficient defects and interfaces, providing obvious merits in both impedance matching and microwave loss. In this review, the recent research progresses on MOF-derived MAMs were profoundly reviewed, including the categories of MOFs and MOF composites precursors, design principles, preparation methods and the relationship between mechanisms of microwave absorption and microstructures of MAMs. Finally, the current challenges and prospects for future opportunities of MOF-derived MAMs are also discussed.

## Highlights

- The metal organic frameworks derived microwave absorption materials (MOF derived MAMs) were systematically reviewed.
- The design principles, preparation methods and effect of microstructures and composites of MOF derived MAMs were discussed.
- The challenges and further research directions of MOF derived MAMs were presented

## Introduction

Microwave technology has been utilized in both civil and military fields, such as wireless communication, medical treatment, scientific research and especially military industry, which are related to national security and social development [1, 2]. Nevertheless, the signals of precision instruments are seriously affected by surrounding microwave radiations [3–5]. Moreover, the excessive microwave emission has been considered as a novel hazardous source of pollution deeply, significantly threating human health [6, 7]. Therefore, designing high-performance microwave absorption materials (MAMs) has become an urgent and indispensable demand [8–10]. Usually, the incident microwave on the MAM surface will be divided into three parts: absorbed, reflected and transmitted waves. To shield microwaves as much as possible, the ideal MAMs should possess good absorption, wide absorption range, light weight and thin thickness. In past decades, many MAMs were designed and investigated, which can be roughly containing three categories: conductive polymers [10–13] (such as polyaniline (PAIN) and polypyrrole (PPy)), conductive carbon matrices [14–16] (such as grapheme (GO) and carbon nanotube (CNT)) and magnetic materials [17, 18] (such as Fe_3_O_4_ and barium ferrite). Although they have many advantages such as cheap, easily available and simple preparation, the mismatched impedance caused by single component severely limited their practical application. The combination of conductive materials and magnetic materials has been proved as an effective method to solve above issue [19], whereas the traditional preparation methods such as electrochemical plating [20], ball-milling [21, 22] and impregnation [23] are only processed at molecular level, which cannot get the MAMs with uniformly component distribution and high porosity. Therefore, designing MAMs with excellent microwave absorption (MA) performance using the effective method is highly desired.

**Fig. 1:**
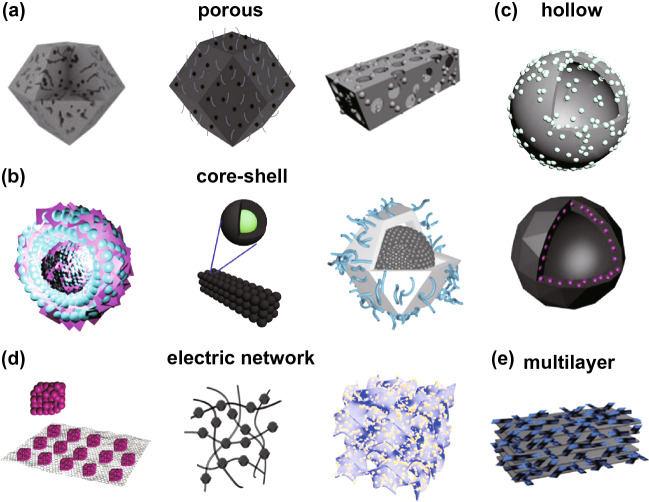
MOF-derived MAMs with different microstructures: **a** porous structure, **b** core–shell structure, **c** hollow structure, **d** electric network, and **e** multilayer structure. Reprinted with permission from Refs. [36–47]

Metal–organic frameworks (MOFs), consisting of the metal ions/cluster centers and organic ligands through coordination interactions, exhibit unique merits including high porosity, large specific surface area and tunable structure [24–26], enabling them huge promising application in various areas such as heterogeneous catalysts[27], energy storage and conversion [28, 29], gas separation [30, 31], sensing [32, 33] and biomedicine [34]. In 2015, the first MOF-derived MAM sample was reported by Liu and *co*-workers [35]. The ZIF-67 was selected as a precursor to obtain porous Co/C nanocomposites after heat treatment; the prepared Co/C nanocomposites exhibited favorable MA performance. Since then, many MOF-derived MAMs with various microstructures have been designed and reported (Fig. 1) [36–47]. Compared with other microwave absorbers, MOF-derived MAMs can maintain the original porous framework and uniform pore distribution and can obtain enhanced magnetic property after appropriate high temperature pyrolysis, promoting the improvement of MA performance [48–50].

Previous works suggested that the pyrolysis temperature, atmosphere and soaking time can have the effect on the composition and structure of MAMs [47, 51]. The pyrolysis temperature mainly affects the graphitization degree of carbon matrix and microstructure of MAMs. The graphitization degree is positively correlated with pyrolysis temperature; higher graphitization degree can provide greater electrical conductivity, which is in favor of the conductivity loss to incident microwave. However, excessive conductivity has negative effects on the MA properties, which can stimulate the induced current when microwave incident was on the surface of MAMs, leading to the enhancement of the microwave reflection and weakening the absorption, resulting in worse MA properties, which is called “skin effect.” On the other hand, surplus conductivity can interact with the incident microwave to form an induced magnetic field in the high-frequency magnetic field, resulting in magnetic energy radiation and negative magnetic loss. Besides, ultrahigh pyrolysis temperature might lead to the collapse of MOF skeletons, resulting in the destruction of porous structures, which is bad for multiple reflection and electromagnetic synergy. For instance, Wang used ZIF-67 with hierarchically porous structure as sacrificial template to obtain porous carbon matrix at three temperatures of 500, 700, and 900 °C [37]. The results showed that the higher temperature could improve the crystallinity, graphitization degree and magnetism of carbon matrix, while the structure was collapsed. Besides, the chemical reaction during the carbonization and chemical constitution of MAMs are also influenced by the pyrolysis atmosphere including argon gas (Ar), nitrogen gas (N_2_), air and mixed gas (Ar/H_2_, N_2_/H_2_). Ar and N_2_ provide the stable condition for the formation of metal nanoparticles. In the case of preparing metal oxides, high-temperature heat treatment is usually carried out directly in air. Besides, the thermal decomposition rate of the material in air is higher than that in Ar and N_2_. As a reducing gas, H_2_ could promote the reduction of metal ions to metal nanoparticles. Especially in the presence of transition metals, the graphitization of carbon can be accelerated effectively, so that Ar/H_2_ and N_2_/H_2_ are also commonly used in some pyrolysis processes.

**Fig. 2:**
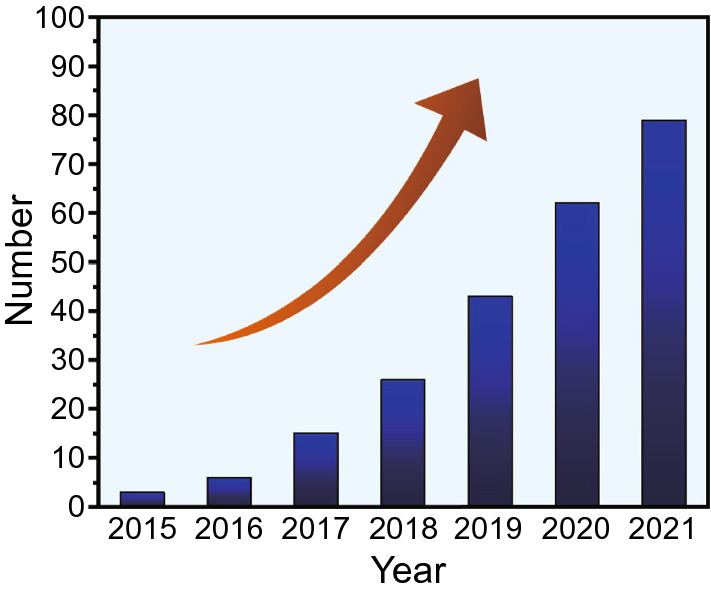
Number of published papers of MOF-based MAMs from 2015 to 2021(Web of science)

MOF-derived MAMs have attracted tremendous research attentions, especially in the last three years, over 150 papers about MOF-derived MAMs have been published (Fig. 2). However, compared with other MAMs materials such as polymer nanocomposites [52, 53], graphene foams [54, 55] and MXenes [56–58], the investigation of MOF-derived MAMs was still in its infant stage; the intrinsic principles of MOF-derived MAMs were still rarely summarized and reviewed. Herein, the recent progress on MOF-derived MAMs is profoundly reviewed. In the initial part of this review, the mechanism of microwaves absorption is introduced. Then, according to the magnetic property of mental center/cluster and number of metallic elements in each center note of MOFs, the MA performance of various MAMs derived from bare MOFs and MOF composites is systematically analyzed to deeply understand the relationship between internal mechanisms and microstructure of MAMs. Moreover, the influences of some crucial parameters in the synthesis of MOF and high-temperature pyrolysis on the whole MA performance are also discussed. In the end, the current challenges are pointed out, and future prospects of MOF-derived MAMs are also given.

## Mechanism of Microwave Absorption

**Fig. 3:**
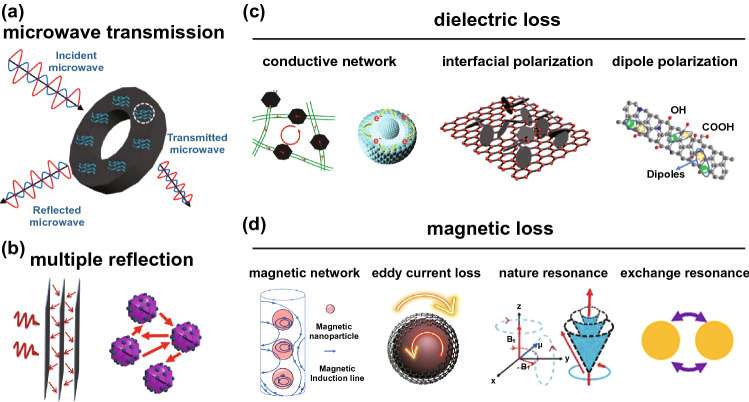
**a** Illustration of the microwave absorption mechanism, including microwave transmission path, **b** multiple reflection inside the MAMs, **c** dielectric loss (conductive network, interfacial polarization and dipole polarization) and **d** magnetic loss (magnetic coupling network, eddy current loss, nature resonance and exchange resonance). Reprinted with permission from Refs. [40, 59–68]

When the microwave reaches the surface of absorber, it will experience three routes: absorption, reflection and transmission routes (Fig. 3a) [59]. The microwave will be reflected by the inner surfaces of the MAMs when microwave enters the inside of MAMs to optimize the internal microwave loss capability of MAMs to absorb microwave (Fig. 3b). However, the reflected and transmitted microwaves could bring secondary pollution. Therefore, one basic principle of designing MAMs is improving the absorption of incident wave to weaken the reflected and transmitted microwave.

Based on the electromagnetic energy conversion principle, there are two main ways to dissipate the microwave, namely dielectric loss and magnetic loss, respectively. The parameters used to represent dielectric loss and magnetic loss are permittivity ($\upvarepsilon$) and permeability ($\mu$), respectively. Both of them consist of real parts (${\upvarepsilon }^{,}$, ${\upmu }^{,}$) and imaginary parts (${\upvarepsilon }^{,,}$, ${\upmu }^{,,}$): the real parts represent the ability of storing electrical or magnetic energy, while the imaginary parts represent the ability of energy dissipation (Eqs. 1 and 2) [69, 70].

1 $$\varepsilon_{r} = \varepsilon^{^{\prime}} - j\varepsilon^{^{\prime\prime}}$$

2 $$\mu_{r} = \mu^{^{\prime}} - j\mu^{^{\prime\prime}}$$

The dielectric loss tangent ($\mathrm{tan}{\updelta }_{\mathrm{E}}$) and magnetic loss tangent ($\mathrm{tan}{\updelta }_{\mathrm{M}}$) are used to describe the dielectric loss capacity and magnetic loss capacity of the MAM, which can be expressed as Eqs. 3 and 4 [71, 72]:

3 $$\tan \delta_{E} = \varepsilon^{^{\prime\prime}} /\varepsilon^{^{\prime}}$$

4 $$\tan \delta_{M} = \mu^{^{\prime\prime}} /\mu^{^{\prime}}$$

The dielectric loss depends on the conductive loss and polarization loss (Fig. 3c). The former is caused by the induced current formed by the migration of free electrons inside the material under electromagnetic field. The MAMs will produce Joule effect when the induced current passed, so that the energy of electromagnetic wave is consumed in the form of heat energy. According to the free electron theory, the ${\varepsilon }^{{^{\prime}}{^{\prime}}}$ shows positive correlation with electrical conductivity (*σ*) (Eq. 5) [73]. The latter is caused by the electromagnetic energy conversion during the polarization relaxation, which mainly includes the interfacial polarization caused by heterointerfaces and dipole polarization resulted from the surface charges, polar molecules and functional groups. According to Debye's law, the relationship between ${\varepsilon }^{^{\prime}}$ and ${\varepsilon }^{{^{\prime}}{^{\prime}}}$ can be expressed by Eq. 6 [74, 75]. The obtained ${\varepsilon }^{^{\prime}}-{\varepsilon }^{{^{\prime}}{^{\prime}}}$ relationship curve is called Cole–Cole curve; the semicircles and upward tail in the curves represent the process of polarization relaxation and conductivity, respectively.

5 $$\varepsilon^{\prime\prime} = \frac{\sigma }{{2\pi \varepsilon_{0} f}}$$

6 $$\left( {\varepsilon^{\prime} - \frac{{\varepsilon_{s} + \varepsilon_{\infty } }}{2}} \right)^{2} + \left( {\varepsilon^{\prime\prime}} \right)^{2} = \left( {\frac{{\varepsilon_{s} - \varepsilon_{\infty } }}{2}} \right)^{2}$$

where ${\varepsilon }_{s}$ is the static dielectric constant, ${\varepsilon }_{\infty }$ is the dielectric constant at infinite frequency, ${\varepsilon }_{0}$ is the dielectric constant in vacuum and $\sigma$ is the conductivity.

Magnetic loss is the process of magnetization and reversal magnetization of magnetic materials in electromagnetic field, which converts electromagnetic energy into heat energy. In the frequency range of 2–18 GHz, the magnetic loss mainly includes hysteresis loss, eddy current loss, natural resonance and exchange resonance (Fig. 3d) [67, 73]. Hysteresis loss is caused by the displacement and rotation of the domain walls during magnetization, while it can be ignored in the weak magnetic field [67]. The eddy current loss can be evaluated by Eq. 7 [47, 66]. It can be proved the magnetic loss is mainly caused by eddy current loss if the value of ${\mu }^{{^{\prime}}{^{\prime}}}{({\mu }^{^{\prime}})}^{-2}{f}^{-1}$ does not vary with $f$. Natural resonance is generated by the intrinsic anisotropic field of the MAM. The natural resonance linewidth is related to damping coefficient [67, 75, 76]. The natural resonance frequency is represented by Eq. 8, so that the natural resonance frequency can be adjusted by regulating the anisotropy constant of the MAM [67]. The effect of exchange resonance needs to be considered when the particles are submicron or nanometer scale, and the exchange formant frequency moves toward high frequency as the particle size decreases [75].

7 $$f_{r} = \frac{{2\gamma K_{{{\text{eff}}}} }}{{M_{s} }}$$

8 $$\mu^{\prime\prime} = \frac{2}{3}\pi \mu_{0} \left( {\mu^{\prime}} \right)^{2} \sigma d^{2} f$$

where $\gamma$, ${K}_{eff}$ and ${M}_{s}$ refer to gyromagnetic ratio, effective anisotropy constant and saturation magnetization, respectively. ${\mu }_{0}$ and $d$ are corresponding to the permeability of vacuum and thickness, respectively.

The MA property of MAM is determined by two factors: impedance matching and attenuation constant. The former determines the amount of microwaves that can enter the absorbers, while the latter is response for the dissipative ability to the incident microwave of the MAMs. Both of them are related to permittivity ($\varepsilon$) and permeability ($\mu$). When the input impedance (${Z}_{in}$) of the MAM is equal to that of the free space (${Z}_{0}$), all of the microwaves arriving at the surface will enter into the MAM, and this phenomenon is called impedance matching. The calculation formulas are as follows:

9 $$Z_{in} = Z_{0} \sqrt {\frac{{ \mu_{r} }}{{\varepsilon_{r} }}} {\text{tanh}}\left( {\frac{2\pi jfd}{c}\sqrt { \mu_{r} \varepsilon_{r} } } \right)$$

10 $$\left| Z \right| = \left| {\frac{{Z_{in} }}{{Z_{0} }}} \right|$$

In addition, the impedance matching can be characterized by a trigonometric function method as well, which is calculated by the following formulas [42, 71, 77]:

11 $$\left| \Delta \right| = \left| {\sinh^{2} \left( {Kfd} \right) - M} \right|$$

12 $$K = \frac{{4\pi \sqrt {\mu_{r} \varepsilon_{r} } \sin \frac{{\delta_{E} + \delta_{M} }}{2}}}{{c \cdot \cos \delta_{E} \cos \delta_{M} }}$$

13 $$M = \frac{{4\mu^{\prime}\cos \delta_{E} \varepsilon^{\prime}\cos \delta_{M} }}{{\left( {\mu^{\prime}\cos \delta_{E} - \varepsilon^{\prime}\cos \delta_{M} } \right)^{2} + \left[ {\tan \left( {\frac{{\delta_{M} }}{2} - \frac{{\delta_{E} }}{2}} \right)} \right]^{2} \left( {\mu^{\prime}\cos \delta_{E} + \varepsilon^{\prime}\cos \delta_{M} } \right)^{2} }}$$

The $\Delta$ function represents the impedance matching degree, and the ideal impedance matching will be achieved when the value of $\Delta$ is close to zero ($\left|\Delta \right|\le 0.4$).

The attenuation constant (*α*) is used to measure the microwave dissipation ability within the MAMs, which relies on the dielectric loss and magnetic loss capacity. Equation 14 is the calculation formula of attenuation constant [75–79]. It can be discovered that the attenuation constant was enlarged with the enhancement of dielectric loss and magnetic loss.

14 $${\upalpha } = \sqrt 2 \frac{\pi f}{c} \times \sqrt {\left( {\mu^{\prime\prime}\varepsilon^{\prime\prime} - \mu^{\prime}{{\varepsilon^{\prime}}}} \right) + \sqrt {\left( {\mu^{\prime\prime}\varepsilon^{\prime\prime} - \mu^{\prime}{{\varepsilon^{\prime}}}} \right)^{2} + \left( {\mu^{\prime}\varepsilon^{\prime\prime} + \mu^{\prime\prime}{{\varepsilon^{\prime}}}} \right)^{2} } }$$

To sum up, the index to evaluate the MA property is the reflection loss (RL) and the effective absorption bandwidth (EAB, RL < -10 dB); 90%, 99% and 99.9% of the electromagnetic wave are lost when the RL is less than − 10, − 20, and − 30 dB, respectively. The RL can be calculated by Eq. 15 [62, 80, 81]:

15 $$RL = 20\left| {{\text{log}}\frac{{Z_{in} - 1}}{{Z_{in} + 1}}} \right|$$

## MAMs Derived from MOFs

According to the number of metallic elements in the center note, a variety of MOFs can be roughly divided in two categories: single-metal-based MOFs and multimetal-based MOFs. Moreover, the magnetic properties of metallic elements play a crucial role in the MA system. In this section, the MAMs derived from single magnetic-metal-based, single nonmagnetic-metal-based MOFs and multimetal-based MOFs are introduced.

### MAMs Derived from Single-Metal-Based MOFs

Usually, MAMs derived from magnetic-metal-based MOFs possess both electrical conductivity and magnetism, providing a convenient way for impedance matching and microwave attenuation. Iron (Fe), cobalt (Co), nickel (Ni) and their alloys all exhibit good magnetic properties, so that Fe-, Co-, and Ni-based MOFs are studied extensively in the microwave absorption field. Different kinds of MAMs derived from single-metal-based MOFs are listed in Table 1.

**Table 1:** MA performance of various MAMs derived from single-metal-based MOFs

MAMs	MOFs	T (°C)	t (h)	Atmosphere	Ratio (wt%)	Reflection loss			Effective bandwidth		Refs
						RL_min_ (dB)	f (GHz)	d (mm)	EAB (GHz)	d (mm)	
Fe_3_C/C	MIL-101(Fe)	650	2	Ar	30	− 39.43	14.00	2.00	–	–	[82]
Fe_3_O_4_@NPC	Fe-MOF	700	5/6	N_2_	40	− 65.5	9.8	3	4.5	3	[83]
Co-C	Co-MOF-74	800	2	N_2_	30	− 62.12	11.85	2.4	–	–	[84]
Co/C	ZIF-67	500	5	Ar	40	− 35.3	5.8	4	5.80	2.5	[35]
Co@NCNT-cube	ZIF-67-cube	500	5	N_2_/H_2_	25	− 52.4	10.8	2.3	4.1	2.3	[85]
Co@NCNT-dodecahedron	ZIF-67-dodecahedron	500	5	N_2_/H_2_	25	− 54.7	15.7	1.6	5.4	1.7	[85]
Co@NCNT-octahedron	ZIF-67-octahedron	500	5	N_2_/H_2_	25	− 53.0	16.8	1.8	6.2	2.0	[85]
Ni/C	Ni(bdc)(ted)_0.5_	500	2	N_2_	40	− 51.8	10.44	2.6	3.48	2.6	[38]
Ni@C	Ni-MOF	600	5	Ar/H_2_	50	− 46.9	6.8	3.5	–	–	[86]
Ni/C	Ni-MOF	600	2	Ar	30	− 57.25	16.1	1.8	5.1	1.8	[41]
Ni@C-ZIF	Ni-ZIF	425	8	Ar	40	− 86.8	7.25	2.7	2.14	2.7	[87]
Ni@C-BTC	Ni-BTC	425	8	Ar	40	− 50.8	~ 8.7	3.2	3.6	3.2	[87]
Ni/C	Ni-MOF	600	2	N_2_	50	− 58	~ 6	5	6.8	1.8	[88]
NPC	ZIF-8	800	2	N_2_	50	− 39.7	8.5	4	4.3	–	[97]
TiO_2_/C	MIL-125(Ti)	800	4	N_2_	40	− 49.6	–	1.6	4.6	1.6	[90]
ZrO_2_/C	UIO-66	800	2	N_2_	50	− 58.7	16.8	1.5	5.5	1.7	[91]

#### Iron-Based MOFs as Precursors

**Fig. 4:**
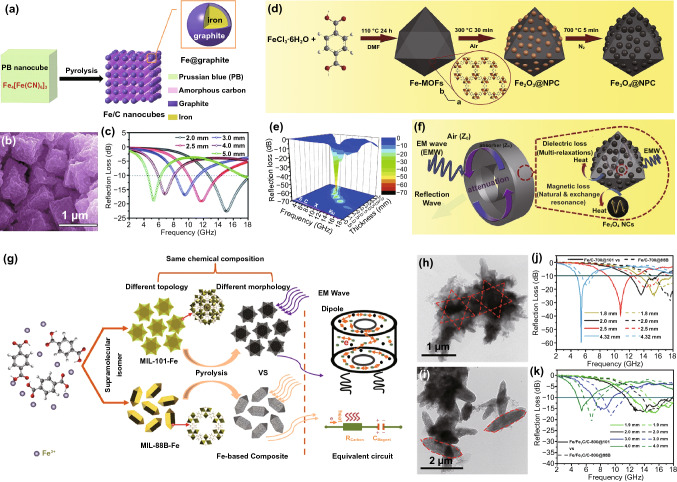
**a** Synthesis method, **b** SEM image and **c** RL-f curves of Fe/C nanocubes. Reprinted with permission from Ref. [92] **d** Schematic diagram of the preparation and **e** RL-f curves of Fe_3_O_4_@NPC. **f** Schematic illustration of the MA mechanism of Fe_3_O_4_@NPC. Reprinted with permission from Ref. [83] **g** Schematic illustration for the synthetic process of the two kinds of FeC nanocomposites. TEM images and RL-f curves of **h, j** FeC-800@101 and **i, k** FeC-800@88B. Reprinted with permission from Ref. [93]

Because of the low cost and excellent magnetic property of iron, Fe-based MOFs are considered as good precursors of MAMs. In 2015, Qiang and *co*-workers used Fe_4_[Fe(CN)_6_]_3_ (PB) as precursors of Fe/C nanocubes (Fe/C), which constituted by the circular nanoparticles with iron as core and graphite as shell (Fig. 4a–b) [92]. The as-prepared Fe/C possessed an RL_min_ of -22.6 dB at 15.0 GHz and EAB of 5.3 GHz (Fig. 4c). Xiang at el. fabricated nanoporous Fe_3_O_4_@C (Fe_3_O_4_@NPC) composites using a novel two-step thermal decomposition method [83]. As shown in Fig. 4d, the Fe_2_O_3_@NPC was obtained after thermal treatment of Fe-MOF at 300 °C in air, the Fe_2_O_3_ was transferred to Fe_3_O_4_ after the subsequent pyrolysis in N_2_, and the organic residues in Fe_2_O_3_@NPC could act as reductant to promote the reduction of Fe^3+^ to Fe^2+^. The Fe_3_O_4_@NPC achieved excellent MA performance with RL_min_ of -65.5 dB at 9.8 GHz as well as EAB of 4.5 GHz (Fig. 4e). The synergy of magnetic and dielectric mechanisms insured favorable impendence matching, and the dual energy attenuation mechanisms provided the conditions of electromagnetic energy transformation (Fig. 4f). Miao et al. prepared a series of MAMs using octahedral MIL-101-Fe and rod-like MIL-88B-Fe as precursors [93]. As shown in Fig. 4g–i, the two precursors were heated under inert atmosphere to obtain FC-T@101 and FC-T@88B (T represented the temperature during the pyrolysis process, *T* = 600, 700, and 800 °C), respectively. The experimental result indicated that FC-600@101 and FC-600@88B did not exhibit effect of RL because of their low permittivity. The higher pyrolysis temperature could obviously enhance the permittivity of Fe-based MOF-derived MAMs. Compared with FC-700@88B and FC-800@88B, the two counterparts derived from MIL-101-Fe presented better MA performance, because the abundant graphite layers could be *in situ* generated during the pyrolysis of MIL-101-Fe and enhance the conductive loss effectively. Particularly, the RL_min_ and EAB of FC-700@101 reached up to −59.2 dB and 6.5 GHz, respectively (Fig. 4j–k). During pyrolysis of Fe-based MOFs in an inert atmosphere, the Fe^2+^ can be converted to Fe_3_O_4_, which can further be transformed to Fe_3_C [94, 95]. However, the presence of Fe_3_C could decrease the magnetism, so that reasonable preparation conditions should be designed to insure the low content of Fe_3_C [92].

#### Cobalt-Based MOFs as Precursors

**Fig. 5:**
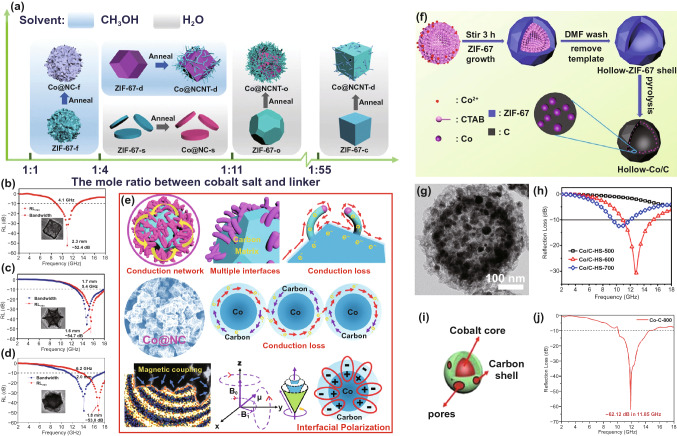
**a** Schematic preparation process of Co@NCNT composites. RL-f curves of the **b** Co@NCNT-cube, **c** Co@NCNT-dodecahedron and **d** Co@NCNT-octadecahedron. **e** Microwave loss mechanism diagram of Co@NCNTs. Reprinted with permission from Ref. [85]. **f** The synthesis route, **g** TEM image and **h** RL-f curves of hollow Co/C microspheres. Reprinted with permission from Ref. [42]. **i** Structure diagram and **j** RL-f curve of C/Co composite. Reprinted with permission from Ref. [84]

It is well known that the whole MA performance of MAM is highly dependent on the morphology of MOF precursor. As a typical Co-based MOF, ZIF-67 presents high potential in MA field because of its ultrahigh porous volume. Moreover, the morphology of ZIF-67 can be precisely controlled by tuning key parameters such as solvent and metal content in the synthesis process; thus, ZIF-67-derived MAMs with varying morphologies have been widely investigated. In 2020, Huang and *co*-workers prepared various ZIF-67 with different morphologies including cube, dodecahedron, octadecahedron, flower and sheet, and then, the corresponding Co@NCNTs were obtained after pyrolysis (Fig. 5a) [85]. As indicated in Fig. 5b-d, the RL_min_ of Co@NCNT-cube, Co@NCNT-dodecahedron and Co@NCNT-octadecahedron were −52.4, −54.7 and −53.0 dB, respectively, which were better than those of Co@NCNTs-flower and Co@NCNTs-sheet, because a plenty of planes provided large surface area of the former three, promoting microwaves enter inside. The MA attenuation mechanism is shown in Fig. 5e, the CNTs on the surface formed connected conductive network and provided conductive loss together with the graphitized carbon matrix; Co nanoparticles exhibited significant magnetic coupling effects and the defects and N-doped sites could be used as polarization centers (Fig. 5e). Besides, the hollow-ZIF-67 was synthesized using a one-step template method, which can be calcinated to receive hollow Co/C microspheres (Fig. 5f–g) [42]. Compared with the normal dodecahedron-like Co/C composites, the hollow Co/C microspheres exhibited better MA performance; the RL_min_ was -31.3 dB at 12.8 GHz when calcined at 600 °C (Fig. 5h). There were mainly two reasons for this experimental phenomenon. Firstly, the higher porous volume was beneficial to the construction of interconnected conductive network and interface, improving the multiple reflection of microwave. Besides, the internal cavity suppressed the interaction among carbon matrix and the incident microwave, restricting the generation of the induced magnetic field and improving the magnetic loss.

Apart from ZIF-67, other Co-based MOFs have also been applied as precursor of MAMs. In 2018, Wang and *co*-workers synthesized Co–C core–shell nanocomposite through carbonization of Co-MOF-74; the prepared Co–C exhibited exceptional MA property with a RL_min_ of  −62.12 dB at the frequency of 11.85 GHz (Fig. 5i–j)[84]. Besides, a flake-assembled jujube pit-shaped C/Co composite by annealing a Co-based MOF displayed an outstanding RL_min_ with  −40.0 dB at 2.8 mm [96].

#### Nickel-Based MOFs as Precursors

**Fig. 6:**
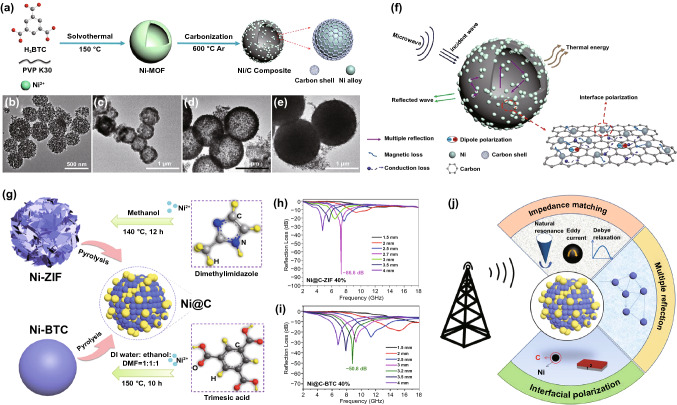
**a** Diagrammatic sketch of the preparation process of Ni/C composites. **b–e** TEM images of four kinds of Ni/C composites. **f** Illustration of MA mechanism for Ni/C composites. Reprinted with permission from Ref. [41]. **g** Schematic illustration of the fabrication process of Ni@C microspheres. RL-f curves of **h** Ni@C-ZIF and **i** Ni@C-BTC. **j** Schematic diagram of MA mechanism for Ni@C microspheres. Reprinted with permission from Ref. [87]

Apart from Fe- and Co-based MOFs, Ni-based MOFs are also important precursors of MAMs. As depicted in Fig. 6a, the Ni/C spheres were directly prepared by heating Ni-MOFs at 600 °C under Ar atmosphere [41]. The mixture of DMF and water was used as an solvent, and the diameter of prepared Ni/C spheres was gradually enlarged with the increase in water content in mixture because the nucleation rate of H_3_BTC in DMF was faster than that in water (Fig. 6b–e). Among these Ni/C spheres, the optimal RL_min_ could reach to −57.25 dB at 16.1 GHz, while the thickness was only 1.8 mm. The great MA property of Ni/C spheres originated from the synergistic effect of multiple reflection, magnetic loss, conductive loss and dipole polarization (Fig. 6f). In 2019, the effect of ligands on the MA property of MOF-derived MAMs was also investigated by Yan and *co*-workers [87]. As shown in Fig. 6g, the dimethylimidazole and trimesic acid were utilized as ligands to prepare Ni-ZIF with spherical-like hierarchical 3D nanostructures and Ni-BTC with smooth and complete microsphere morphology, respectively. The experimental results revealed that Ni@C-ZIF and Ni@C-BTC both exhibited superior MA properties in the frequency of 2 to 18 GHz (Fig. 6h-i). In particular, the RL_min_ of Ni@C-ZIF reached to -86.8 dB at 13.2 GHz, which was mainly attributed to N-contained ligands resulting in the improvement of interfacial polarization. As shown in Fig. 6j, the appropriate magnetic loss and dielectric loss endowed the MAM with excellent impedance matching, and the multiple reflection and interfacial polarization also played significant roles in the microwave dissipation. Moreover, Zeng et al. used dimethylimidazole and pyromellitic acid as the mixed ligands to prepare Ni-based MOF as precursor of Ni@C nanocomposite in 2021 [86]. The RL_min_ of Ni@C was obtained when the pyrolysis temperature was 600 °C (−46.9 dB at 3.5 mm).

#### Single Nonmagnetic-Metal-Based MOF-Derived MAMs

Recently, some nonmagnetic-metal-based MOFs have been attempted as precursors of MAMs. Due to the absence of magnetic loss medium, the selection of precursors and the design of the experimental process are both based on the adjustment of dielectric constant and microstructure. At present, the main research mainly includes the following.

Due to the relative low melting point (420 °C) and boiling point (908 °C), Zn-based MOFs are increasingly concentrated. The Zn element in Zn-based MOFs could convert to ZnO when the calcination temperature is higher than 550 °C [97]. ZnO, a typical polarization semiconductor with low electrical conductivity and wide bandgap, has been extensively used in adjusting MA property [98–100]. Besides, the carbon-reduced Zn metal would start to be vaporized when the pyrolysis temperature exceeded 800 °C, leading to the formation of amorphous structure to increase the porosity of the MAMs [89]. In 2018, Wu calcined ZIF-8 at three different temperatures (700, 800 and 900 °C) and the products named NPC700, NPC800 and NPC900, respectively [97], in which NPC800 owned the largest RL_min_ (−39.7 dB) because of its appropriate porosity and well-maintained skeleton. However, the MW properties of Zn-based MOF-derived MAMs are greatly restricted to the absence of magnetism. So far, rarely studies have been focused on MAMs derived from pure Zn-contained MOF; however, introducing magnetic components in Zn-contained MOF and combining Zn and magnetic metals as mixed centers to form multimetal MOF as MAMs precursor have attracted tremendous research attention in microwave absorption field.

**Fig. 7:**
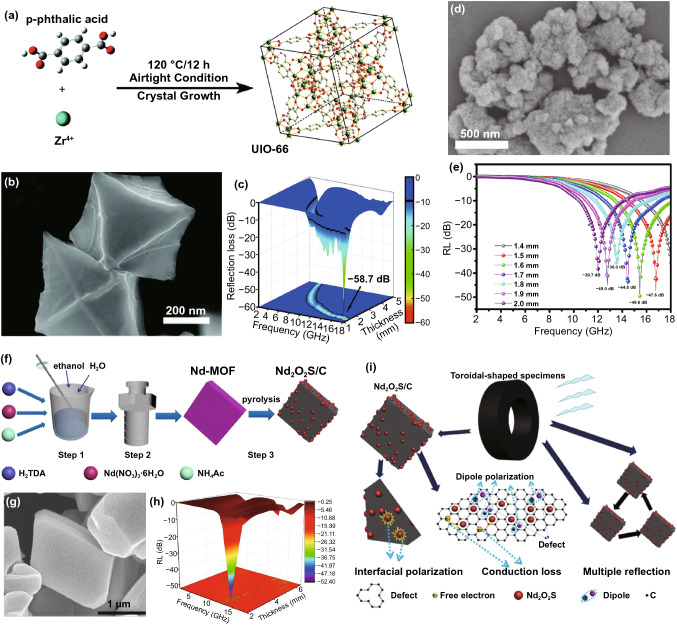
**a** Schematic illustration of diagram and crystal structure of UIO-66. **b** TEM image and **c** 3D RL-f curves of ZrO_2_/C. Reprinted with permission from Ref. [91]. **d** TEM image and **e** RL-f curves of TiO_2_/C. Reprinted with permission from Ref. [90]. **f** Diagrammatic sketch of the preparation process, **g** SEM image, **h** 3D RL-f curves and **i** the MA mechanism of Nd_2_O_2_S/C. Reprinted with permission from Ref. [103]

As typical ceramic dielectric materials, ZrO_2_ and TiO_2_ show great application potentials in MA field due to their excellent chemical stability, environmental friendliness and mass storage [101]. Moreover, the ZrO_2_ and TiO_2_ in the nanocomposites played a key role in achieving good impedance matching because their relatively low electrical conductivity could neutralize the excessive conductive loss of the graphitized carbon [101]. Therefore, the Zr- and Ti-based MOFs grasped an increasing attention in MA field because the excellent thermal stability enables them to form metal oxides rather than metallic state during carbonization [101]. For instance, Zhang and *co*-workers obtained ZrO_2_/C composites after the carbonization of UIO-66 with excellent MA performance; the optimized RL_min_ and EAB were  −58.7 dB (16.8 GHz, 1.5 mm) and 5.5 GHz (Fig. 7a-c), respectively [91]. Ma and co-workers used MIL-125(Ti) as the precursor to obtain TiO_2_/C nanocomposites with an RL_min_ of  −49.6 dB and EAB of 4.6 GHz, respectively [90]. Figure 7d–e shows the TEM images and RL-f curves of TiO_2_/C nanocomposites. Except for the enhanced impedance matching, the natural porous structure of MIL-125 also made nonnegligible contribution to the internal reflection of TiO_2_/C. The rare-earth oxides such as CeO_2_ and Nd_2_O_3_ also exhibited improved MA properties through combining with carbon materials [102]. Shen and *co*-workers synthesized an ingenious rhombic Nd_2_O_2_S/C nanocomposite by direct thermal treatment of Nd-MOF (Fig. 7f–g) [103]. The RL_min_ of Nd_2_O_2_S/C carbonized at 800 °C reached -52.3 dB at 2.56 mm thickness (Fig. 7h). In addition to the conductive loss originated from the graphitized carbon matrix, the interfacial polarization caused by many heterogeneous interfaces between Nd_2_O_2_S and porous carbon and the dipole polarization originated from carbon defects and oxygen-containing functional groups also played crucial roles in enhancing MA capacity (Fig. 7i).

**Fig. 8:**
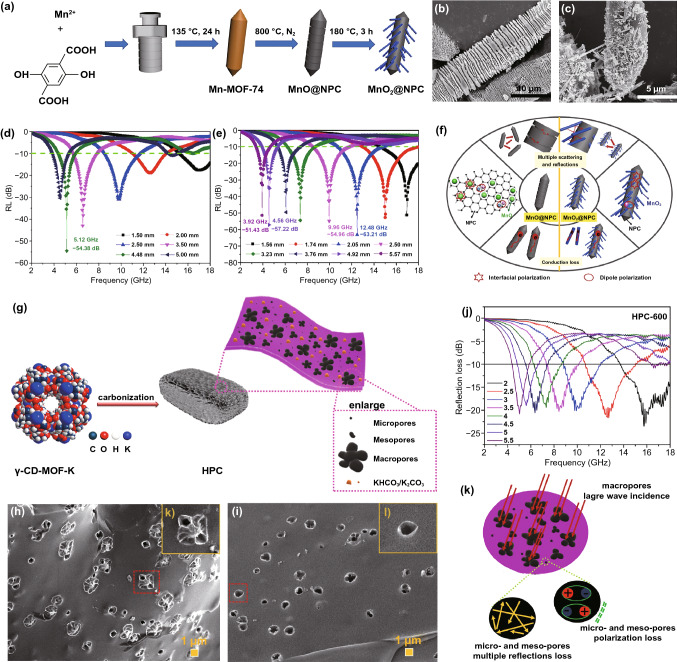
**a** Schematic illustration of diagram of MnO@NPC and MnO_2_@NPC. TEM image and 3D RL-f curves of **b, d** MnO@NPC and **c, e** MnO_2_@NPC. **f** Schematic illustration of MA mechanisms for MnO@NPC and MnO_2_@NPC. Reprinted with permission from Ref. [108]. **g** Preparation process diagram of γ-CD-MOF-K-derived HPC. TEM images of **h** HPC-600 and **i** HPC-900. **j** RL-f curves and **k** MA mechanism of TiO_2_/C. Reprinted with permission from Ref. [109]

Nowadays, manganese oxides have been increasingly studied in the fields of MA owing to their environmental friendliness, abundant resources and unique physical and chemical properties [104]. MnO, a semiconductor with wide bandgap of 3.6 eV, has low conductivity and weak attenuation ability, exhibiting excellent wave-transparence [105, 106]. MnO_2_ is also a peculiar metallic semiconductor with great metal-like electrical conductivity and dielectric property [107]. Liu’s group synthesized MnO@NPC and MnO_2_@NPC with Mn-MOF-74 as precursors [108]. The caterpillars-like MnO@NPC was prepared by directly carbonizing Mn-MOF-74 at 800 °C, and the hedgehog-like MnO_2_@NPC was obtained after the subsequent hydrothermal reaction of MnO@NPC (Fig. 8a-c). Both MnO@NPC and MnO_2_@NPC achieved surprising MA performance, especially all the RL_min_ of MnO_2_@NPC was higher than −50 dB in S, C, X, and Ku bands (Fig. 8d-e). In this system, the significant anisotropy and high aspect ratio of MnO_2_@NPC were conducive to the formation of efficient conductive network, and the dipole polarization was also enhanced by the additional dipole centers resulted from oxidation of MnO@NPC, which skillfully optimized the MA performance of MnO_2_@NPC (Fig. 8f).

Some other metal-centered MOFs have also been attempted as precursors for MAMs due to their unique properties. For instance, Zhang et al. fabricated ingenious hierarchical porous carbon (HPC) materials using γ-CD-MOF-K as carbonizing template with K^2+^ acted as activator in the formation of HPC (Fig. 8g) [109]. The HPC-600 was obtained by directly pyrolyzing γ-CD-MOF-k at 600 °C, and the HPC-600-900 was fabricated by calcining HPC-600 at 900 °C subsequently. As shown in Fig. 8h and i, the flower-like pore structure and ordinary circular pore structure were observed on the surface of HPC-600 and HPC-600–900, respectively. The HPC-600 showed effective MA property with a RL_min_ of -23.5 dB and EAB of 4.3 GHz, while the MA property of HPC-600–900 was poor (Fig. 8j). Pore structure and pore size distribution had great influence on their impendence matching and electromagnetic energy attenuation, in which the flower-like pore could bring in more heterogeneous interfaces and the abundant micro- and mesopores were in favor of the multiple reflection loss and polarization loss (Fig. 8k). Besides, some familiar metal ions have also been tried as the centers of MOF precursors. For instance, Ji’s group reported a MgO/C composite derived from CPO-27-Mg [110]. The RL_min_ of MgO/C was −14.93 dB at the 2.0 mm and the EAB achieved 4.9 GHz.

### MAMs Derived from Multimetal-Based MOFs

For single magnetic-metal-based MOFs, the type and amount of metal ions are relatively fixed, so the permeability and permittivity of MAMs derived from them can only be adjusted by changing the heating temperature. Therefore, using multimetal-based MOFs as precursors can effectively avoid this problem, because the electromagnetic parameters of MAMs could be effectively adjusted by changing the ratio of different metals. Besides, the morphologies of multimetal-based MOFs can be easily tuned when metal ions with different valence states share the mental center/cluster, because the different coordination numbers and geometries can influence the growth rate of building units [111, 112]. The multimetal-based MOF precursors can be divided into these basic three types: (i) multi-magnetic-metal-based MOFs, (ii) multi-nonmagnetic-metal-based MOFs and (iii) mixed-metal-based MOFs. The MA properties of the MAMs derived from above multimetal-based MOFs are summarized in Table 2.

**Table 2:** MA performance of various MAMs derived from multimetal-based MOFs

MAMs	MOFs	T(°C)	t (h)	Atmosphere	Ratio (wt%)	Reflection loss			Effective bandwidth		Refs
						RL_min_ (dB)	f (GHz)	d (mm)	EAB (GHz)	d (mm)	
CoNi@C	CoNi-MOFs	800	2	Ar	–	− 44.8	10.7	3.2	–	–	[113]
NiCo/CNR@CNT	NiCo-MOF-74	700	2	Ar	5	− 58.8	14.0	2.2	6.5	2.2	[114]
Ni_1-x_Co_x_@ C	Ni-Co-MOF	600	5	Ar	25	− 59.5	6.0	4.5	4.9	2.2	[66]
Fe_0.8_Ni_0.2_@C	Ni/Fe-MOF	750	–	Ar	30	− 71.3	10.3	3.1	5.3	–	[115]
Co/Ni/C	CoNi-MOF-71	600	1	Ar	40.0	− 49.8	12.2	2.6	7.6	2.6	[76]
FeCoNi@C	FeCoNi-MOF-74	700	2	N_2_	38	− 64.75	15.44	2.1	8.08	2.47	[116]
CNCo	Co/Zn-BMZIF	600	2	N_2_	–	− 51	-	1.65	5.24	1.9	[117]
CoZn/C	Co/Zn-BMZIF	500	3	Ar	–	− 59.7	4.9	4.5	5.3	3	[118]
Co/ZnO/C microrods	CoZn-MOF cuboids	700	1	N_2_	30	− 52.6	12.1	3.0	5.8	2.5	[119]
BPCNs	Co/Zn-ZIFs	650	3	N_2_	15	− 45.2	6.32	5.0	5.7	2.5	[120]
ZnO/Fe_3_C/C	Fe^III^-MOF-5	700	2	N_2_	40	− 30.4	14.5	1.5	4.96	1.5	[95]
Ni@C@ZnO	NiZn-MOF	600	5	Ar	25	− 55.8	–	2.5	4.1	–	[40]
Co@CNTs-3	CoCd-MOF-11	1000	2	Ar	20	28.3	–	1.8	3.0	2.2	[121]
Co@CNTs-2	CoZn-MOF11	1000	2	Ar	20	76.7	–	2.0	6.24	2.0	[121]
Co@C@MnO	CoMn-MOF-74	700	2	Ar	20	64.4	13.5	2.6	6.7	4.0	[104]

#### Multi-magnetic Metal MOFs as Precursors

**Fig. 9:**
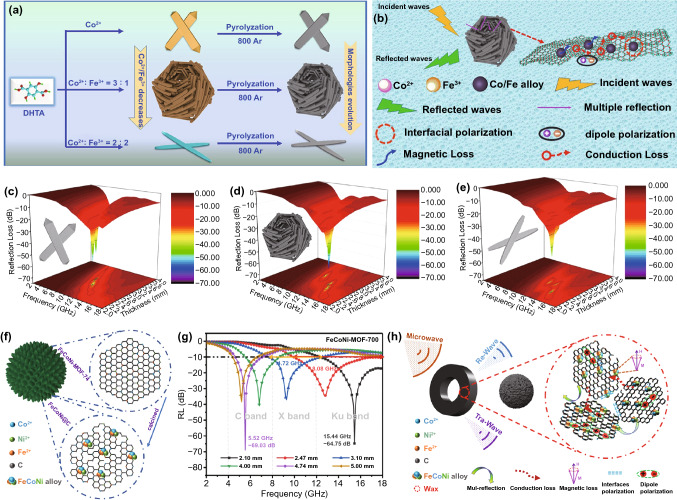
Schematic illustration of **a** preparation process and **b** MA mechanism for CoFe@C composites with rod-like, nest-like and sheet-like structures. **c–e** 3D RL-f curves of three kinds of CoFe@C composites. Reprinted with permission from Ref. [122]. **f** Schematic illustration of preparation process, **g** RL-f curves and **h** MA mechanism of hollow FeCoNi@C nanocomposites. Reprinted with permission from Ref. [116]

One confront challenge is that the impedance mismatch of MOF-derived MAMs was caused by the sufficient permittivity and inadequate permeability. Using two or more magnetic metal ions as mental center/cluster is a promising method to moderate this issue because the various magnetic mental and alloy nanoparticles can be generated during pyrolysis process. In 2020, Wang and *co*-workers prepared three different CoFe-MOF-74 nanocomposites by simply adjusting the molar ratio of Co/Fe [122]. As shown in Fig. 9a, the samples shown rod-like, nest-like and sheet-like morphologies when the Co/Fe was 4:0, 3:1 and 2:2, respectively. The CoFe@C composites derived from the above samples all exhibited great attenuation abilities of MW, which originated from the magnetic loss of Co/Fe alloy, conductive loss of graphitized carbon, multiple reflection supplied by porous structure and polarization loss of heterogeneous interfaces and defects (Fig. 9b). The MA properties of these three CoFe@C nanomaterials were −55.5 dB at 10.6 GHz, −61.8 dB at 12.7 GHz, −47.5 dB at 13.9 GHz, and the EAB was 5.4, 9.2 and 7.4 GHz, respectively (Fig. 9c-e). The relatively better performance of hierarchical nest-like CoFe@C benefited from its porous structure and reasonably constitute. Similarly, Ji’s group synthesized a CoNi@C sample using CoNi-MOF as precursor, obtaining a RL_min_ of −43.7 dB with a low thickness of 1.7 mm [123]. In 2019, the hollow FeCoNi@C nanocomposite was synthesized through roasting the flowered spherical MOF containing Fe, Co, and Ni multimetal ions at high temperature (Fig. 9f) [116]. The molar ratio of each component in the FeCoNi alloy was varied with the carbonization temperature, significantly affecting the lattice parameters and the magnetic properties of FeCoNi alloy. The experimental results indicated that FeCoNi and CoFe magnetic alloys coexisted in FeCoNi@C nanocomposites when the calcination temperature was 700 °C, which achives the optimal performance with RL_min_ of −64.75 and −69.03 dB at 15.44 and 5.52 GHz, respectively (Fig. 9g). The magnetic loss of FeCoNi alloy played an important part in the MW attenuation mechanism, which enhanced the impendence matching and microwave attenuation ability (Fig. 9h).

#### Multi-nonmagnetic Metal MOFs as Precursors

**Fig. 10:**
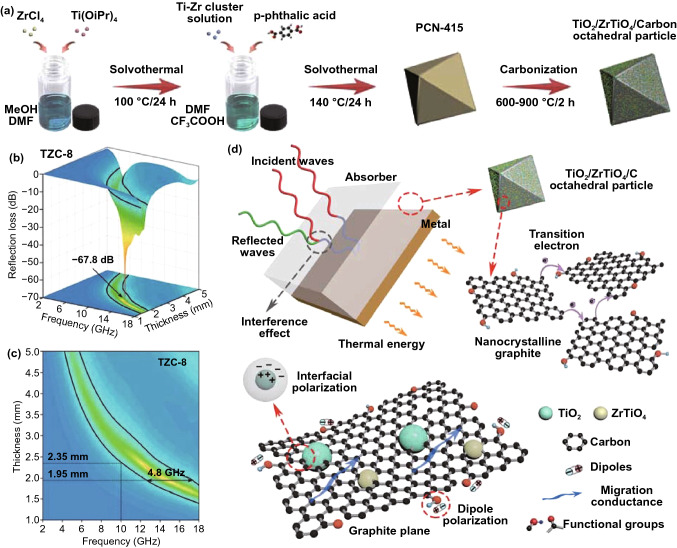
**a** Diagrammatic sketch of the preparation. **b** 3D RL-f curves,** c** 2D RL projection mapping and **d** schematic illustration of the MA mechanism of TiO_2_/ZrTiO_4_/carbon nanocomposites. Reprinted with permission from Ref. [101]

It is worthy to attempt that we use multi-nonmagnetic metal MOFs with enhanced interfacial polarization and high porosity as precursors to prepare MAMs. Typically, Qiao and *co*-workers prepared TiO_2_/ZrTiO_4_/carbon nanocomposites using PCN-415 (TiZr-MOFs) as precursors [101]. The PCN-415 precursors were prepared through two-step solvothermal reactions, and the TiO_2_/ZrTiO_4_/carbon nanocomposites were obtained after high temperature treatment (Fig. 10a). The experimental results showed that the TiO_2_/ZrTiO_4_/carbon nanocomposites exhibited the best MA performance with a carbonization temperature of 800 °C, and the RL_min_ and EAB of TiO_2_/ZrTiO_4_/carbon reached −67.8 dB and 4.8 GHz, respectively (Fig. 10b-c). For comparison, the authors fabricated ZrO_2_/C and TiO_2_/C with UIO-66 and MIL-125 as precursors under the same preparation conditions, respectively. However, the MA properties of those two MAMs were poor. The factors contributing to the above phenomenon were the effective adjustment ability for impedance matching and rich phase interfaces of TiO_2_/ZrTiO_4_/carbon. The MA mechanism of TiO_2_/ZrTiO_4_/carbon is depicted in Fig. 10d: firstly, the transition electron in nanocrystalline graphite provided conductive attenuation; secondly, the polarization loss originated from rich heterogeneous interfaces and functional groups further enhanced the MA property; finally, the TiO_2_ and ZrTiO_4_ optimized the natural impedance mismatching of carbon matrix, providing a necessary premise for microwave to enter TiO_2_/ZrTiO_4_/carbon. More importantly, the MA ability of TiO_2_/ZrTiO_4_/carbon was even better than many MAMs derived from magnetic metal MOFs, proving the potential of multi-nonmagnetic metal MOFs in microwave absorption.

#### Mixed Metal MOFs as Precursors

**Fig. 11:**
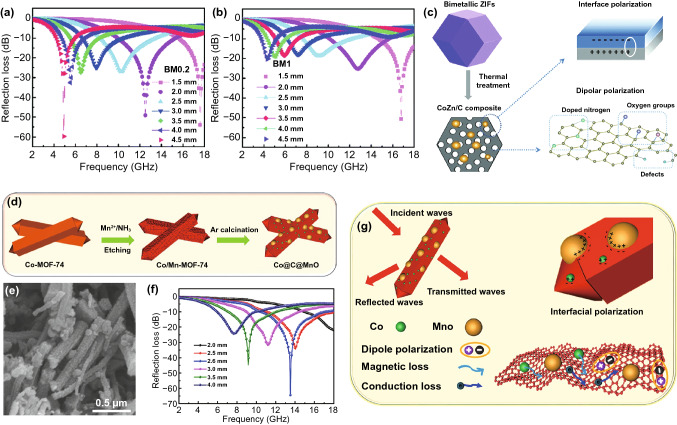
RL-f curves of CoZn/C with the Zn/Co of **a** 0.2 and **b** 1. **c** Schematic illustration of the synthesis of CoZn/C composites. Reprinted with permission from Ref. [118]. **d** Schematic illustration of preparation process, **e** SEM image, **f** RL-curves and **g** MA mechanism diagram of Co@C@MnO nanorod. Reprinted with permission from Ref. [104]

At present, there are also many works used mixed metal MOFs as precursors of MAMs. One of the common nonmagnetic metal sources is Zn^2+^, which can transfer into ZnO with excellent dielectric properties and Zn which can provide plentiful pores to enhance the multiple reflection. In 2021, Wei and *co*-workers pyrolyzed a ZIF containing both Co and Zn to obtain CoZn alloy/carbon(CoZn/C) composites [118]. The Co^2+^ and Zn^2+^ were existed simultaneously and dispersed uniformly in this ZIF because they could coordinate with 2-methylimidazole ligands with the same structure. The graphitization, pore size distribution and surface area characteristics could be precisely controlled by adjusting the molar ratio of Zn to Co. Compared with ZIF-67, the pyrolysis products of bimetal ZIF presented lower pore size distribution and higher surface area because of the volatilization of Zn. The RL_min_ of the CoZn/C composites with the Zn/Co of 0.2 was −59.7 dB and Zn/Co of 1 was  −50.7 dB (Fig. 11a and b). In addition to the conductive loss and magnetic loss, the abundant heterogeneous interfaces brought about interface polarization, the doped nitrogen, oxygen groups and defects supplied sufficient dipole polarization when microwave enters the CoZn/C composites. Besides, the abundant pores created conditions for the multiple reflections, which contributed to the MA attenuation effectively (Fig. 11c). When the pyrolysis temperature is between 550 and 800 °C, the zinc element in zinc-based MOF mainly exists in the form of ZnO. Wang designed a yolk-shell Ni@C@ZnO microsphere with an open gap between the core and shell by directly calcining Ni-Zn-MOF [40]. The RL_min_ of Ni@C@ZnO reached -55.0 dB and the EAB covered 4.1 GHz, which could attribute to the increasing interface polarization and optimization of impedance matching.

In order to increase interfacial polarization, dipole polarization or optimize impedance matching, some other metal ions have also been attempted to be used in the center of MOF together with magnetic metal ions to prepare precursors of high-performance MAMs. Qiu et al. fabricated CoMn-MOF-74 nanomaterials by the etching method [104]. The Mn^2+^ coordinated with the ligands in Co-MOF-74 to form CoMn-MOF-74, and the Co@C@MnO nanorods were obtained after calcination of CoMn-MOF-74 (Fig. 11d-e). The RL_min_ was −61.8 dB at 13.5 GHz, and the EAB was 6.7 GHz (Fig. 11f). The Co nanoparticles were uniformly distributed in the carbon matrix, which could produce strong magnetic loss and conductive loss. Besides, there were many interfaces between Co, C and MnO, effectively enhancing the interface polarization (Fig. 11g). In 2021, Zhang and *co*-workers synthesized a novel Co/CoO/SrCO_3_@C nanocomposite using CoSr-bimetal MOF as precursor [124]. This product exhibited great MA performance with RL_min_ of -39.51 dB at 2 mm and EAB of 2.55 GHz at a relatively thin thickness of 1 mm, in which the interwoven nanoparticles and hollow nanotubes played a significant part on the MA progress.

## MAMs Derived from MOF Composites

To further optimize the electromagnetic parameters of MAMs, combining MOFs with other materials with great electrical or magnetic conductivity as carbonized precursors is also a popular method. Compared with pure MOFs as precursors, the preparation process of MOF composites is slightly more complex, but it can adjust the electromagnetic parameters more specifically and effective. There are three main methods to form MOF composites: (i) introducing magnetic nanoparticles, (ii) constructing MOF and low-dimensional material hybrids and (iii) designing core–shell structure. In this section, three methods are introduced to deeply understand the mechanism of microwave absorption to guide the future relative research.

### Introducing Magnetic Nanoparticles

The impedance mismatch is a problem to face for many MOF-derived MAMs. An effective way to solve the problem is increasing magnetic nanoparticles of MAMs to improve the magnetic loss capacity. There are two ways to achieve this goal: incorporating magnetic nanoparticles into MOF and mixing metal salts with precursor solution of MOF. In Table 3, we make a list of the MA performance of the MAMs as mentioned above.

**Table 3:** MA performance of various MAMs derived from metal-doped MOFs

MAMs	MOF composites	T (°C)	t (h)	Atmosphere	Ratio (wt.%)	Reflection loss			Effective bandwidth		Refs
						RL_min_ (dB)	f (GHz)	d (mm)	EAB (GHz)	d (mm)	
Co/C	Co NPs/ZIF-67	700	5	Ar	25	− 30.31	11.03	3	4.93	3	[125]
Ni/NiO/Cu@C	Cu3(btc)_2_/Ni^2+^	700	2	Ar	10	− 38.1	–	3.2	-	–	[126]
Co/ZrO_2_/C	Co(NO_3_)_2_/NH_2_-UIO-66	800	2	N_2_	50	− 57.2	15.8	3.3	11.9	–	[127]
ZnO-Co@N/C	ZnCo_2_O_4_@ZIF-67	500	2	Ar	30	− 62.7	5.98	4.05	5.75	2	[72]
Co_3_ZnC-Co@N/C	ZnCo_2_O_4_@ZIF-67	650	2	Ar	30	− 67.97	5.6	4.22	5.23	1.97	[72]
CoFe alloys@ZnO@C	Fe_2_O_3_@ZnCo-MOF	650	2	Ar	30	− 44.13	–	5.0	5.84	5.84	[70]

#### Doping Magnetic Nanoparticles

**Fig. 12:**
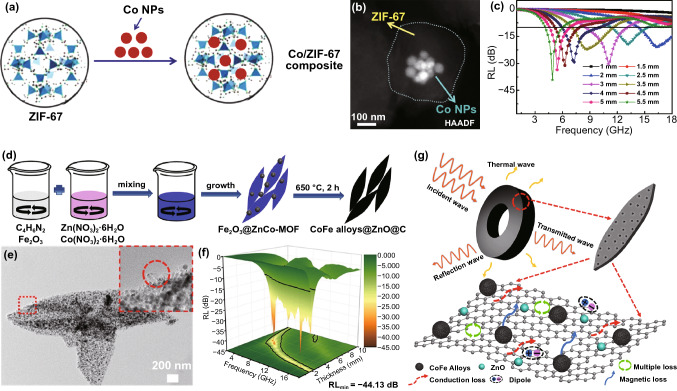
**a** Schematic diagram of the fabrication and **b** HAADF image of Co NPs/ZIF-67, **c** RL-f curves of Co/C nanocomposites. Reprinted with permission from Ref. [125]. **d** Schematic illustration of experimental procedures **e** TEM and **f** RL-f curves of CoFe alloys@ZnO@C composites. **g** Schematic illustration of the MA mechanism of CoFe alloys@ZnO@C. Reprinted with permission from Ref. [70]

To improve the magnetic property of MAMs, incorporating magnetic metal nanoparticles (NPs) or metallic oxide into MOF precursors is a direct method. In 2015, a novel Co-Fe/NPC nanomaterial was prepared by the thermal decomposition of Fe_3_O_4_ NP-modified ZIF-67 (IONP@ZIF-67) [128]. Although the preparation process of Fe-Co/NPC was relatively complex, including the preparation of Fe_3_O_4_ NPs, encapsulation of Fe_3_O_4_ with DNAA, and the synthesis of IONP@ZIF-67 and Fe-Co/NPC, the MA performance of Fe-Co/NPC nanomaterials was quite good with RL_min_ of -21.7 dB with a thickness of 1.2 mm and a broad EAB of 5.8 GHz. Liu embedded Co NPs into ZIF-67 to act as the precursors of Co/C nanocomposites (Fig. 12a) [125]. Co NPs were firstly modified with PVP and then mixed with Co(NO_3_)_2_ to produce Co NPs/ZIF-67. As shown in Fig. 12b, the Co NPs/ZIF-67 maintained the dodecahedron structure and the Co NPs were fully encapsulated in ZIF-67. The Co/C nanocomposites shown improved impedance matching and thus an excellent MA ability with RL_min_ of -30.31 dB at 11.03 GHz and EAB of 4.93 GHz (Fig. 12c). Similarly, Wu’s group synthesized CoFe alloys@ZnO@C composites by directly carbonizing Fe_2_O_3_@ZnCo-MOF at 650 °C (Fig. 12d) [70]. The obtained CoFe alloys and ZnO nanocomposites were evenly distributed in carbon matrix, and some carbon nanotubes appeared in the surface (Fig. 12e). The optimized RL_min_ achieved -40.63 dB at a thickness of 2.2 mm and −44.13 dB at 5.0 mm (Fig. 12f). The CoFe alloys mainly contributed magnetic loss of this MAM, and the presence of ZnO balanced the high conductivity of graphitized carbon and than improved the impedance matching performance. Besides, the heterogeneous interface, defects and polarization groups also greatly contributed to the attenuation of microwave (Fig. 12g).

#### Mixing Metal Salts with Precursors Solution of MOF

**Fig. 13:**
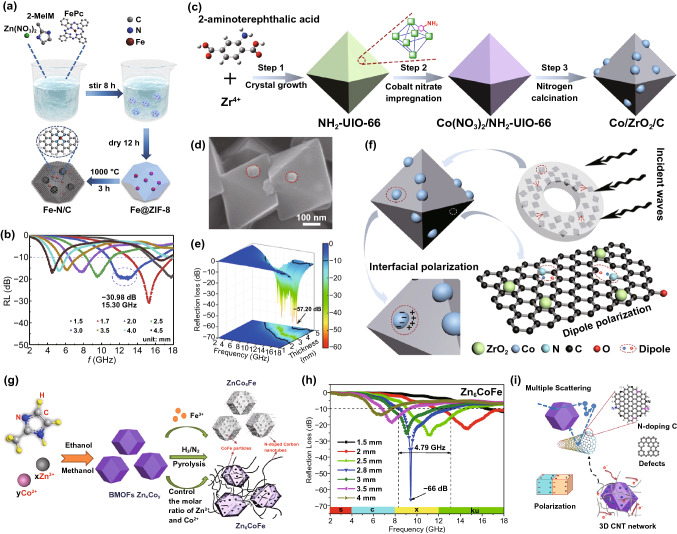
**a** Diagrammatic sketch of the synthesis and **b** RL-f curves of Fe–N/C. Reprinted with permission from Ref. [129]. **c** Schematic illustration of the preparation, **d** SEM image, **e** 3D RL-f curves, **f** MA mechanism of Co/ZrO_2_/C octahedrons. Reprinted with permission from Ref. [127]. **g** Schematic diagram of synthesis, **h** RL-f curves and **i** schematic representation of MA mechanism of Zn_x_Co_y_Fe@C@CNTs. Reprinted with permission from Ref. [130]

Compared with directly doping magnetic nanoparticles, mixing metal ions with precursor solution of MOFs is more simple and efficient method to optimize magnetic loss and further promote the impedance matching. Yu prepared a novel Fe–N/C nanocomposite using Fe@ZIF-8 as precursor through one-pot method [129], in which the phthalocyanine (FePc) was added simultaneously with 2-MeIm and Zn^2+^ to *in situ* get Fe@ZIF-8 composite, which was followed by the high-temperature calcination to obtain Fe–N/C (Fig. 13a). During the pyrolysis process, the Zn^2+^ transformed into Zn NPs and then vapored, whereas the Fe^2+^ was reduced to Fe NPs. The Fe–N/C achieved a MA property with the optimized RL_min_ of −30.98 dB at a low thickness of 1.7 mm (Fig. 13b).

The central atoms of electron-donating groups (such as –NH_2_ and –COOH) are electronegative, which can effectively capture the metal ions and form the coordination bonds. Zhang successfully synthesized Co/ZrO_2_/C octahedrons by the carbonization of Co(NO_3_)_2_-impregnated NH_2_-UIO-66 [127]. The Co^2+^ was coordinated with N of –NH_2_ to form Co(NO_3_)_2_/NH_2_-UIO-66; the pyrolysis product displayed the primitive morphology with Co NPs embedded in the surface (Fig. 13c-d). The Co/ZrO_2_/C possessed the excellent MA performance with RL_min_ of  −57.2 dB at 15.8 GHz and EAB covered as wide as 6.5 GHz (Fig. 13e). The introduced Co NPs provided the magnetic loss to enhance the impendence matching and attenuation capacity. Besides, the number of heterogeneous interfaces, defects and polarization groups, which benefited to polarization loss, was also improved (Fig. 13f).

Besides, solution infiltration is also a common approach to add metal ions into MOFs. The metal ions could permeate into the channel of MOFs through the capillary force at the orifice and the metal nanoparticles would be embedded into carbon matrix after pyrolysis. In 2020, Yan impregnated the CoZn-MOF precursors with FeCl_3_ solutions; the corresponding Zn_x_Co_y_Fe@C@CNTs were obtained after calcination in the mixture of H_2_ and N_2_ (Fig. 13g) [130]. In the heating process, the cavity could be generated because of the volatilization of Zn, which promoted the encapsulation of Fe to form CoFe alloy and catalyzed the growth of CNTs. As shown in Fig. 13h, the RL_min_ of Zn_x_Co_y_Fe@C@CNTs reached -66 dB and the EAB covered the whole X band. The great MA performance originated from the multiply scattering, polarization loss originated from N-doping C, defects and many interfaces, conductive loss of 3D CNT networks and the magnetic loss of CoFe alloy (Fig. 13i). In 2019, Huang and *co*-workers prepared Ni/NiO/Cu@C nanocomposites using Cu_3_(btc)_2_/Ni^2+^ as precursors [126]. The Ni^2+^ was ground into the ordered pores of Cu_3_(btc)_2_ with assist of ethanol flow, resulting in the incorporation of Ni NPs into the Cu@C nanocomposites to produce NiO NPs on the surface after the high-temperature calcination. Compared with Cu@C nanocomposites, the MA property of Ni/NiO/Cu@C was obviously improved with RL_min_ of -38.1 dB at 3.2 mm when the filler loading of Ni/NiO/Cu@C in paraffin matrix was merely 10 wt%.

### Loading on Low-Dimensional Materials

Generally, the ideal MAMs should have conductive networks with good conductive loss; however, most MOF-derived MAMs only can possess good conductivity rather than conductive networks, which seriously limit their further application in MA field. Therefore, designing MOF-derived MAMs containing low-dimensional conductive carbon materials and porous magnetic component is considered as an efficient approach to solve the above issue. The conductivity of the composites can be improved and the surface electron transfer also could be accelerated. Meanwhile, the defects can be generated at the interface and surface, resulting in the increase in dielectric loss. Generally, high graphitization materials can be obtained by the high-temperature treatment of some carbon-rich materials such as conductive carbon materials, biomaterials, MXene and g-C_3_N_4_. Besides, some other materials with large specific surface area, electrical or dielectric properties are also worth to try. A series of works published in the area of MAMs derived from MOF hybrids with low-dimensional materials are presented in Table 4.

**Table 4:** MA performance of various MAMs derived from MOF hybrids with low-dimensional materials

MAMs	MOF composites	T (°C)	t (h)	Atmosphere	Ratio (wt%)	Reflection loss			Effective bandwidth		Refs
						RL_min_ (dB)	f (GHz)	d (mm)	EAB (GHz)	d (mm)	
carbon-cotton/Co@NPC	cotton/ZIF-67	700	2	N_2_	25	− 60.0	8.48	2.55	4.4	1.65	[131]
cotton fiber-Co/C/CNT	ZIF-67@cotton fiber	900	2	H_2_/Ar	10	− 53.5	7.8	2.9	8.02	2.0	[132]
Co/C-RGO	ZIF-67@RGO	500	6	Ar	6	− 52	9.6	4.1	7.72	3.2	[133]
CoZn@NCNTHS/GO	ZnCo-MOF/GO	700	2	Ar	6	− 47.31	−	1.5	4.01	1.5	[65]
NiFe@C @GO	NiFe-PB@GO	700	2	N_2_	30	− 51	7.7	2.8	6.2	2.2	[43]
rGO-CoFe@C	GO-ZnCo-MOF	900	1.5	Ar	10	− 36.08	13.01	3.0	5.17	3.0	[77]
CNT-CoFe@C	CNT-ZnCo-MOF	900	1.5	Ar	10	− 40.00	9.86	3.0	5.62	2.0	[77]
Co–C/MWCNTs	ZIF-67/MWCNTs	700	4	Ar	15	− 48.9	−	2.99	−	–	[68]
MWNCTs@CoO	MWCNTs@ZIF-67	700	5	Ar	10	− 50.2	−	1.84	−	–	[134]
Co/Co_3_O_4_/C@CNT	ZIF-67/CNTs	500	3	N_2_	20	− 30	14.2	1.5	7.8	3.0	[135]
Co/SiC NWs	MOFs/SiC NWs	500	6	Ar	10	− 40	−	5	4.84	2.0	[136]
CoO/SiC NWs	MOFs/SiC NWs	400	1	air	10	− 47	9.32	3	5.92	2.0	[136]
NC-Co_3_O_4_/CP	A-CP@ZIF-67	350	2	air	40	− 41.38	7.32	2.3	−	–	[137]
FeNi@CNT/CNRs	MA/FeNi-MIL-88B	700	2	N_2_	20	− 47.0	−	2.3	4.5	1.6	[138]
CoNC/CNTs/C	CoZn-ZIF-L	700	2	N_2_	15	− 44.6	5.20	4.7	4.5	1.5	[63]
Co/Si/C/N	P-ZIF-67@PDSDA	700	5	Ar	30	− 50.9	17.0	1.9	5.72	1.9	[39]

#### Loading on Conductive Carbon Materials

Common conductive carbon materials include graphene oxide (GO), carbon nanotube (CNT), carbon nanofiber (CNF), etc., which are considered to the ideal template for constructing lightweight MAMs due to their high strength, large specific surface area and high conductivity [139–141]. There are a large number of active sites (such as –OH and –COOH groups) in the surface of GO and CNT, which can absorb metal ions through electrostatic interaction to form MOFs in situ. For instance, Zhang and *co*-workers produced Co/C-rGO composites by calcining GO-wrapped ZIF-67 (Co@ZIF-67) at high temperature [133]. The corresponding RL_min_ reached –52 dB at 9.6 GHz, and the EAB was 7.72 GHz. Xu proposed a novel method by using CoNi-BTC/rGO as precursors to produce pomegranate-like CoNi@NC/rGO-600 nanocomposites [49], in which the CoNi@NC/rGO-600 exhibited outstanding MA performance with a broad EAB of 6.7 GHz and a RL_min_ of –68.0 dB. The authors also served CoNi@NC-600 derived from CoNi-BTC as a comparative reference, but its MA property was poorer than that of CoNi@NC/rGO-600 obtained in the same condition; the main reason for this phenomenon was that GO provided a smooth conductive network.

**Fig. 14:**
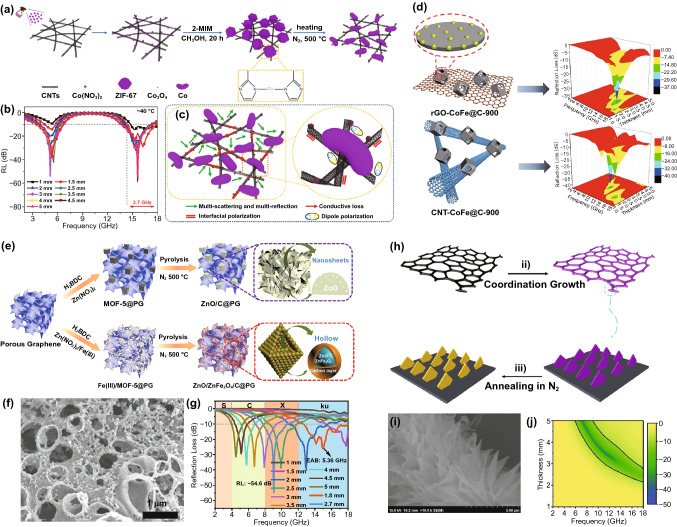
**a** Schematic illustration of the synthesis **b** RL-f curves and **c** MA mechanism of Co-based MOF-derived carbon/CNTs. Reprinted with permission from Ref. [135]. **d** Schematic illustration of preparation and RL-f curves of rGO-CoFe@C and CNT-CoFe@C. Reprinted with permission from Ref. [77]. **e** Schematic illustration of the synthesis of ZnO/C@PG and ZnO/ZnFe_2_O_4_/C@PG. **f ** SEM image and **g** RL-f curves of ZnO/ZnFe_2_O_4_/C@PG. Reprinted with permission from Ref. [45]. **h** Schematic illustration the synthesis. **i** SEM image and **j** 2D RL projection mapping of Co_3_O_4_/CF. Reprinted with permission from Ref. [142]

In 2021, Fang and *co*-workers loaded ZIF-67 on CNTs to serve as the precursors of hierarchical Co@C/CNTs [135]. As shown in Fig. 14a, the Co^2+^ in ZIF-67 mainly be reduced to Co nanoparticles during the pyrolysis process, and a little part were oxidized to Co_3_O_4_. The experimental results revealed that the as-prepared Co@C/CNTs exhibited strong MA performance both at room temperature and low temperature of  40 °C. The RL_min_ was achieved −57 dB at 4.5 mm and −30 dB at a thin thickness of 1.5 mm (Fig. 14b), in which dielectric loss and magnetic loss were provided by the interconnected CNT network and Co-contained magnetic-particles, respectively. Actually, the morphology also plays a key role in MA performance of MAMs (Fig. 14c). In 2020, the effect of the introduction of different dimensional carbon (rGO or CNT) matrix was studied by Wang and *co*-workers. They synthesized rGO-CoFe@C and CNT-CoFe@C nanocomposites using GO and CNT as carriers to grow ZnCo MOF in situ, following by embedding Fe^3+^ in the pores of ZnCo-MOF and calcining under Ar atmosphere (Fig. 14d) [77]. The RL_min_ and EAB of rGO-CoFe@C reached −36.08 dB and 5.17 GHz, respectively. However, CNT-CoFe@C had a better performance with RL_min_ up to −40.00 dB and EAB of 5.62 GHz. The reasons for this phenomenon were that CNTs that owned high aspect ratio and the P electrons in carbon atoms were conjugated to form large π bonds, which made it easier to form three-dimensional conductive network than rGO.

In addition to one-dimensional and two-dimensional materials, three-dimensional materials also have great application potential in the field of microwave absorption due to their porous structure, interconnected networks and abundant interfaces. Song et al. used ingenious three-dimensional porous graphene (PG) as template to grow MOF-5 and Fe(III)-MOF-5 and treated at high-temperature to prepare ZnO/C@PG and ZnO/ZnFe_2_O_4_/C@PG materials (Fig. 14e) [45]. The pyrolysis products still maintained the nanoscale hollow structure, which increases the propagation path of electromagnetic wave in the MAM (Fig. 14f). Furthermore, ZnO/ZnFe_2_O_4_/C@PG had a stronger magnetic loss than ZnO/C@PG due to the introduced Fe(III), so that exhibited more impressive MA performance with a RL_min_ of −54.6 dB at 9.04 GHz and EAB of 5.36 GHz (Fig. 14g). Liu’ group loaded ZIF-67 on the surface of lightweight carbon foam (CF) to act as the precursors of Co_3_O_4_/CF (Fig. 14h) [142]. During the high-temperature pyrolysis process, the Co^2+^ transferred into Co_3_O_4_ sheet embedded on the carbon matrix, which benefited the dual electromagnetic energy attenuation mechanisms (Fig. 14i). The RL_min_ of Co_3_O_4_/CF was -46.58 dB at 10.72 GHz, and the EAB was as broad as 5.4 GHz (Fig. 14j).

#### Loading on Biomaterials

**Fig. 15:**
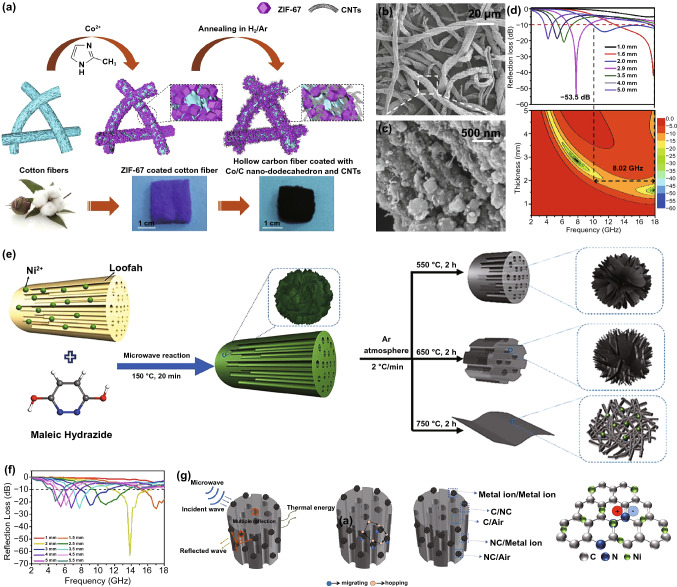
**a** Schematic illustration of the preparation **b, c** SEM images and **d** RL-f curves and corresponding 2D RL-f mapping of carbon fiber/Co@C/CNTs. Reprinted with permission from Ref. [132]. **e** Schematic illustration of the synthesis process of Ni/NC/C-T composites. **f** RL-f curves and **g** MA mechanism of Ni/NC/C-T-650. Reprinted with permission from Ref. [146]

Due to the environmental friendliness, easy availability and low cost, many natural biomaterials have been used as carbon sources to prepare ultra-lightweight high-performance MAMs in recent years [143–145]. Despite their high dielectric constant, the biomaterial-derived porous carbon materials are lack of magnetic loss system and cannot achieve appropriate impendence matching [145]. Researchers often recombine biomaterials with other functional materials, especially magnetic materials, to explore superior MA properties. Many researchers have attempted to load MOFs onto biomaterials to serve as the precursors of MAMs, and this approach has been proved promising. One of the most common biomaterials is biomass cotton; Zhao et al. selected cotton/ZIF-67 as precursor to produce porous and fibrous structure of carbon-cotton/Co@NPC composite [131]. Compared with the product calcined by ZIF-67 and cotton alone, the MA performance of the carbon-cotton/Co@NPC was significantly improved, the optimal performance includes RL_min_ of -51.2 dB and EAB of 4.4 GHz at only 1.65 mm. Besides, Yang and *co*-workers fabricated hollow carbon fiber/Co@C/CNTs by pyrolysis ZIF-67/cotton fiber in H_2_/Ar (Fig. 15a–c) [132], in which the interwoven fibers and CNTs promoted the formation of excellent conductive network. The ultra-lightweight hollow carbon fiber/Co@C/CNTs showed excellent MA ability with a RL_min_ of −53.5 dB at 7.8 GHz and an EAB of 8.02 GHz (Fig. 15d).

**Fig. 16:**
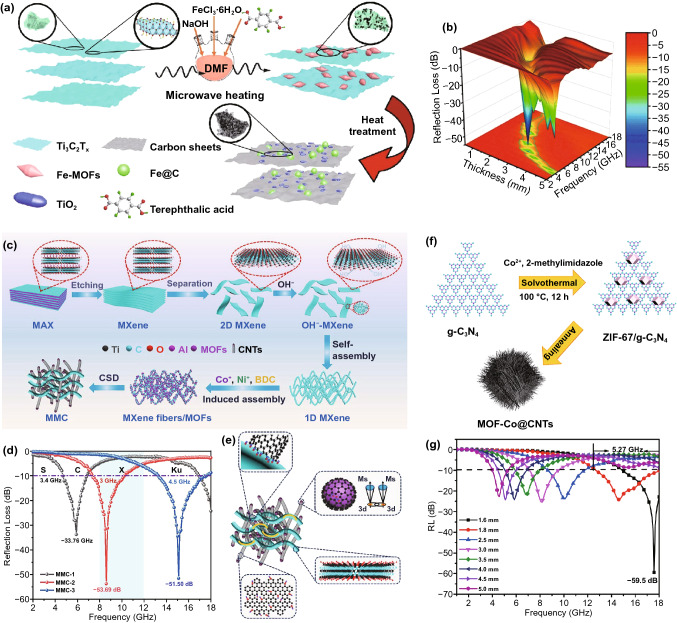
**a** Schematic illustration of the synthesis and **b** 3D RL-f curves of Fe&TiO_2_@C. Reprinted with permission from Ref. [156]. **c** Schematic description for preparation **d** RL-f curves and **e** MA mechanism diagram of MXene fibers@MOF-derived CNTs nanocomposites. Reprinted with permission from Ref. [59] **f** Diagrammatic sketch of the synthesis route and **g** RL-f curves of ZIF-67/g-C_3_N_4_. Reprinted with permission from Ref. [157]

Other natural biomaterials, such as wood [147], walnut shell [148], and spinach [149], are also attracted the attention of researchers due to particular structure. As depicted in Fig. 16e, the Ni-MOF was uniformly grown on the surface of loofah by a microwave method, and three kinds of Ni/NC/C-T (T referred to pyrolysis temperatures) with different shapes were fabricated by carbonization (Fig. 15e) [146]. The Ni/NC/C-650 exhibited the best MA property (RL_min_ = −63.1 dB, EAB = 5.12 GHz), which was mainly due to the particular construction, high porosity, abundant interfaces, plentiful dipoles and the synergistic effect of magnetic loss and dielectric loss (Fig. 15f–g). Besides, Fei et al. designed ultra-lightweight aerogels of ZIF-67/bacterial cellulose (BC)-derived CNF@Co/C aerogels with high porosity and extremely low density, and the CNF@Co/C aerogels displayed high EMI SE of 56.07 dB at ultra-low density [150].

#### Loading on MXene

As a novel class of two-dimensional material, metal carbides and nitrides (MXene) have attracted an increasing research attention in the field of microwave absorption due to their excellent metallic conductivity, unique layered structure, tunable active surface and outstanding electrical conductivity [151–154]. Han and *co*-workers designed MXene/Co-ZIF and MXene/Ni-ZIF precursors by coating Co-ZIF and Ni-ZIF on the interlayer of MXene by the electrostatic self-assembly method [155]. The obtained two MAMs, labeled as MXene/Co-CZIF and MXene/Ni-CZIF, both achieved outstanding MA performance: the optimal RL_min_ of MXene/Co-CZIF was -60.09 dB at 7.36 GHz with an ultrawide EAB of 9.3 GHz and the measured RL_min_ of MXene/Ni-CZIF was −64.11 dB and the EAB was 4.56 GHz, in which the layered structure of MXene greatly enhanced the internal multiple dissipation of microwave to enhance the whole MA performance. Similarly, Deng et al. prepared Fe&TiO_2_@C composites with MXene/Fe-MOFs as precursors by rapid microwave-assisted heating reaction and high-temperature carbonization [156]. The Fe and TiO_2_ NPs sandwiched between the MXene-derived carbon nanosheets to from a parallel multilayer structure, which greatly contributed to the attenuation of microwave and impedance matching of MA process (Fig. 16a). The Fe&TiO_2_@C exhibited a RL_min_ of −51.8 dB and a broad EAB of 6.5 GHz with a low matching thickness of 1.6 mm (Fig. 16b).

Generally, the layer number of MXene can be regulated by adjusting the etching conditions. The single-layer MXene always exhibits higher electrical conductivity than multilayer MXene [152]. Wang et al. designed 3D hierarchical NiCo transition metal oxide@MXene composites by electrostatical self-assembly and high-temperature treatment, which exhibited great MA performance with RL_min_ of -67.22 dB at 1.70 mm and a broad 6.72 GHz with a low filler loading of 5 wt%. Wu synthesized novel MXene fibers@MOF-derived CNTs nanocomposites (MMC) by annealing MXene fibers/CoNi-MOF (MM) precursors [59]. As indicated in Fig. 16c, the one-dimensional MXene fibers were obtained by self-assembly of two-dimensional MXene sheets under a strong alkaline environment. Then, the MM-x (*x* = 1, 2, 3, representing the multiple of the added amount of BDC, Co^2+^ and Ni^2+^ to the initial dose) was prepared by embedding CoNi-MOF on one-dimensional MXene fibers in suit and the MMC-x was obtained after carbonization. The MMCs exhibited excellent MA properties especially the RL_min_ (−51.6 dB) of MMC-2 and EAB (4.5 GHz) of MMC-3 (Fig. 16d). The MA mechanism is depicted in Fig. 16e, the layered porous structure promoted the multiple reflection of incident microwave and optimized impedance matching, the evenly distributed CoNi NPs provided the eddy current and natural resonance, and the defects, interfaces and interwoven 3D conductive networks equipped the MMC with excellent dielectric loss.

#### ***Loading on g-C***_***3***_***N***_***4***_

Because of possessing abundant negative charge atoms, N, graphitic carbon nitrides (g-C_3_N_4_) display the strong ability to capture cations, which effectively promotes the combination with MOFs. However, the g-C_3_N_4_, which is unstable at high temperatures, can be decomposed at 600 °C and almost transferred into cyanide fragments when the temperature is above 700 °C. During the thermal decomposition process, the carbon materials are etched to increase porosity and the doped N can serve as polarization sites to enhance dipole polarization. Zhu synthesized MOF-Co@CNTs with ZIF-67/g-C_3_N_4_ as precursors by the carbonization method [157]. The g-C_3_N_4_ was prepared by pyrolyzing melamine, and the ZIF-67 was uniformly embedded on g-C_3_N_4_ (Fig. 16f). During the carbonization process, the organic skeleton and Co^2+^ of ZIF-67 were converted into graphitized carbon and Co NPs, respectively. Furthermore, the Co NPs could catalyze g-C_3_N_4_ into CNTs growing on the surface of ZIF-67 to form conductive network, promoting electron transfer to enhance the conductivity loss. The MOF-Co@CNTs exhibited an excellent RL_min_ of −59.5 dB with a layer thickness of only 1.6 mm and a broad EAB of 5.27 GHz at 1.8 mm (Fig. 16g). Besides, Jin and *co*-workers used porous rod-shaped g-C_3_N_4_ as template to grow ZIF-67 and then obtained micron-sized Co-doped porous carbon-based MAM (CN/CoC) at high temperature [158]. The RL_min_ and EAB of CN/CoC were −37.21 dB at 12.22 GHz and 6.45 GHz.

#### Loading on Other Low-Dimensional Materials

Conductive polymers, including polypyrrole, polythiophene and polyaniline,also draw an increasing research attention in the field of microwave absorption due to their excellent electrical conductivity [10, 159, 160]. For example, Sun and *co*-workers prepared a chain-like polypyrrole (PPy) aerogel ornamented with ZIF-67-derived Co/C composites (Co/C@PPy aerogel) using the self-assembled polymerization method [161]. The Co/C nanoparticles was mixed with pyrrole monomer and aged in mixture solvent of deionized water and anhydrite, and then aerogels were obtained after dialysis. The Co/C nanoparticles were uniformly dispersed on the PPy chains, which constructed excellent three-dimensional conductive network to provide conductive loss. The Co/C@PPy aerogel exhibited a high RL_min_ of −44.76 dB at 2 mm.

**Fig. 17:**
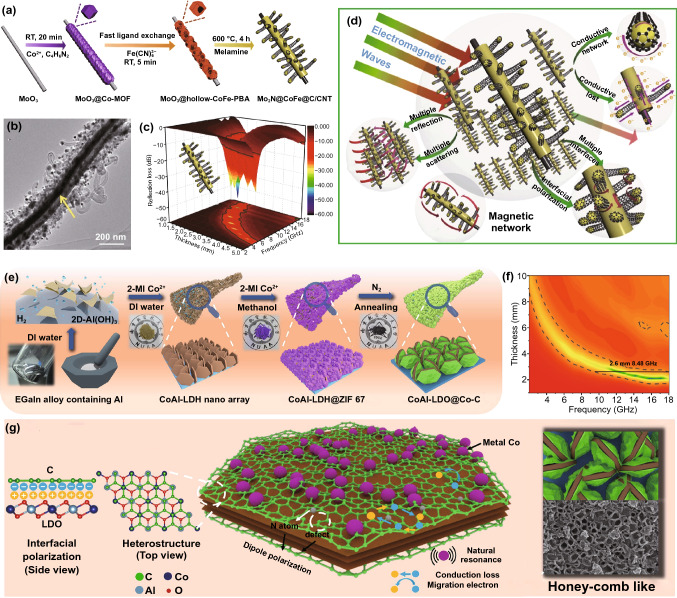
**a** Schematic representation of the synthesis, **b** TEM image, **c** 3D RL-f curves and **d** MA mechanism of Mo_2_N@CoFe@C/CNT. Reprinted with permission from Ref. [164]. **e** Schematic description for preparation route, **f** 2D RL-f mapping and **g** MA mechanism of CoAl-LDO@Co-C. Reprinted with permission from Ref. [165]

Some oxides, nitrides and sulfides of transition metals (such as ZnO and Mo_2_S) with outstanding dielectric performance are also stimulated the research interest [162, 163]. Che’s group produced core–shell MoO_3_@hollow-CoFe-PBA through in situ self-assembly and ligand exchange reaction of ZIF-67, than obtained the final Mo_2_N@CoFe@C/CNT composites by calcining with melamine (Fig. 17a) [164]. The CoFe nanoparticles acted as the catalyst of graphite carbon and CNTs which derived from melamine to grow in situ on the surface of Mo_2_N, constructing a novel “tubes on rods” structure (Fig. 17b). Impressive MA capability was achieved which exhibited a RL_min_ of −53.5 dB with a bandwidth of 5.0 GHz at a low thickness of 2 mm (Fig. 17c). The microwave dissipation capacity of this MAM included the following four ways: interface polarization of heterogeneous interfaces, efficient electron transfer paths of C/CNTs and Mo_2_N nanorods, magnetic loss of CoFe nanoparticles and multiple reflection created by layered core–shell structure (Fig. 17d).

Layered double hydroxides (LDH), lamellar compounds constructed by positively charged lamellae and negatively charged anions, are considered as a kind of promising MAMs because of their abundant active sites and chemical stability [165, 166]. Zhang and *co*-workers designed ingenious CoAl-LDH@ZIF-67 using liquid metal alloy-driven unique 2D hydroxide as template, in which the regular Co in CoAl-LDH served as the nucleation and growth sites to assist ZIF-67 embedded on CoAl-LDH stably [165]. The CoAl-LDO@Co-C nano-arrays were synthesized successfully after high-temperature treatment (Fig. 17e). The CoAl-LDO@Co-C achieved a relatively broad EAB of 8.48 GHz at a thickness of 2.6 mm, which surpassed a large amount of MOF-derived MAMs (Fig. 17f). The MA mechanism is shown in Fig. 17g, abundant heterojunction interfaces, defects and N atoms caused efficient polarization loss, graphitized carbon and uniformly dispersed Co magnetic loss provided great conduction loss and magnetic loss, and the honey-comb like structure supplied a favorable condition for the multiply reflection.

Some fibrous or rod-like materials with special properties are also studied as templates to load MOF precursors. For instance, silicon carbide nanowires (SiC NWs) with lightweight, large specific surface area and high stability were used as templates to grow ZIF-67 [136]. The pyrolysis product Co@C/SiC displayed a RL_min_ of −47 dB and a EAB of 5.92 GHz, which mainly attributed to the multiple reflection loss derived from the great connectivity of SiC NWs and reasonable synergy of dielectric loss and magnetic loss.

### Constructing Core–shell Structure

**Fig. 18:**
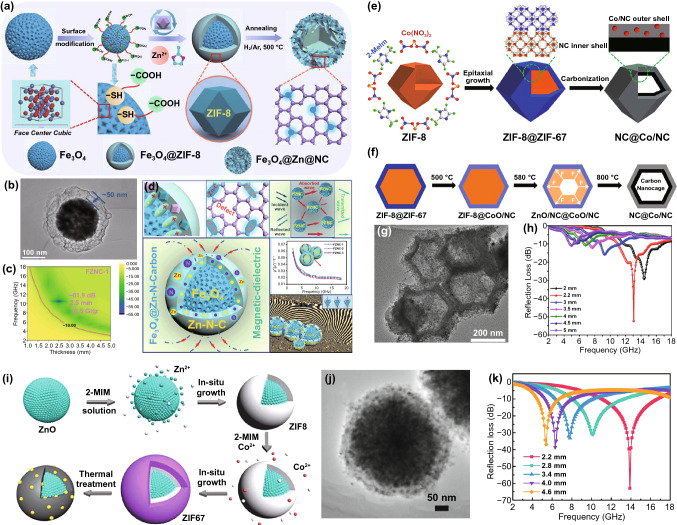
**a** Diagrammatic sketch of the synthesis, **b** TEM image, **c** 2D RL-f mapping and **d** schematic diagram of MA mechanism of Fe_3_O_4_@Zn-N-Carbon. Reprinted with permission from Ref. [168]. **e** Schematic illustration of formation process, **f** synthetic mechanism, **g** TEM image and **h** RL-curves of NC@Co/NC carbon nanocages. Reprinted with permission from Ref. [172]. **i** Schematic illustration of the synthesis, **j** TEM image and **k** RL-curves of ZnO@carbon/Co_3_ZnC. Reprinted with permission from Ref. [169]

Core–shell structure can assimilate the divided chemical and physical properties from both core and shell, easily overcoming the limitation of single component through various combinations of conductive materials and magnetic materials [167]. Table 5 lists the typical MAMs derived from core–shell MOF composites and their MA properties. For instance, Huang prepared a novel Fe_3_O_4_@Zn-N-Carbon (FZNC) nanocomposite as a magnetic-dielectric synergy MAM [168]. As shown in Fig. 18a–b, Fe_3_O_4_ microsphere was modified with carboxyl group to provide deposition sites for Zn^2+^, and ZIF-8 was further generated in situ to synthesis core–shell Fe_3_O_4_@ZIF-8. Then, the ZIF-8 shell transformed into Zn-N-Carbon layer coating the Fe_3_O_4_ magnetic core to obtain core–shell FZNC nanocomposite during the pyrolysis process. The FZNC achieved an optimized MA performance with a RL_min_ of −61.9 dB at 13.1 GHz and a extremely wide EAB of 11.5 GHz (Fig. 18c). The special structure constructed rich interfaces, polarization groups and defects, the graphitized carbon provided a pathway for electron migration, and the ingenious FeO-Fe_3_O_4_ heterojunction interface improved the capacity of trapping electron/hole carriers and constructed the interfacial energy barrier (Fig. 18d).

**Table 5:** MA performance of various MAMs derived from core–shell MOF composites

MAMs	MOF composites	T (°C)	t (h)	Atmosphere	Ratio (wt%)	Reflection loss			Effective bandwidth		Refs
						RL_min_ (dB)	f (GHz)	d (mm)	EAB (GHz)	d (mm)	
ZnO@C/Co_3_ZnC	ZnO@ZIF8@ZIF67	500	2	Ar	40	− 62.9	–	2.2	5.5	2.2	[169]
FeCo@C@CNGs	FeCo PBAs@PDA	600	2	Ar	50	− 67.8	15.8	2.0	5.3	2.0	[170]
N-Ni-Co_x_S_y_/Ni_x_S_y_@C	Ni-ZIF67/S@CMC	900	2	N_2_	25	− 48.3	11.7	2.0	3.95	1.5	[60]
SnO_2_/Co_3_Sn_2_@C	CoSnO_3_@MOF	600	3	Ar	–	− 56.2	6.8	4.5	4.8	2.5	[171]
SnO_2_/Co_3_Sn_2_@Air@C	H-CoSnO_3_@MOF	600	3	Ar	–	− 56.8	11.0	3.0	6.1	2.5	[171]
CF@C/Co	CF@ZIF-67	600	6	N_2_	20	− 71.95	–	1.78	6.25	1.71	[48]

It should be noted that the ZIF-67 could nucleate and grow on the ZIF-8 because of their same crystal structures. In 2020, Liu designed a extraordinary N-doped carbon nanocage with NC as inner shell and Co/NC as outer shell (NC@Co/NC) using the core–shell ZIF-8@ZIF-67 as precursor (Fig. 18e) [172]. The difference in thermal stability between ZIF-8 and ZIF-67 was the key to the successful preparation of NC@Co/NC carbon nanocage. In the carbonization process, ZIF-67 decomposed firstly to form the stable Co/NC shell, which produced an outward adhesion force to inhibit the inward contraction of NC derived from ZIF-8 (Fig. 18f-g). The micro-meso-macropores improved impedance matching and reflection loss of NC@Co/NC, which exhibited outstanding MA property with a RL_min_ of -52.5 dB at 13.1 GHz and EAB of 4.4 GHz (Fig. 18h). Feng synthesized carbon-coated ZnO with Co_3_ZnC nanoparticles (ZnO@carbon/Co_3_ZnC) embedded in the carbon matrix for microwave absorption [169]. The ZnO was used as template, and the ZIF-8 in the middle layer and ZIF-67 in the outermost layer grew on ZnO; then, the ZnO@carbon/Co_3_ZnC nanocomposites were successfully synthesized after the high-temperature treatment (Fig. 18i–j). The RL_min_ of ZnO@carbon/Co_3_ZnC nanocomposites was dramatically enhanced to −62.9 dB at 7.8 GHz, and the EAB was up to 5.5 GHz (Fig. 18k).

## Summary and Outlook

Recently, MOF-derived MAMs have made a great progress in microwave absorption field, acting as promising candidates for high-performance MAMs owing to their superior properties. In this review, various MOF-derived MAMs are systematically introduced and discussed. The MOF precursors can be classified into two major categories: pure MOFs and MOF composites. Numerous MOF-derived MAMs exhibited high MA performance with the RL_min_ less than −50 dB and the EAB greater than 4 GHz, proving huge application potential value of MOF-derived MAMs in the field of microwave absorption. In brief, the MA properties of MAMs mainly depend on the impedance matching and attenuation constant, which both relate to the capacity of dielectric loss and magnetic loss; we detailedly analyze the adjustment methods of the electromagnetic parameters from the aspects of component and structure design and summarize some promising methods to improve the MA capacity of MAMs. For MOF-derived MAMs, heat treatment condition is one of the most important factors to improve microwave attenuation performance; this paper analyzes the influence of pyrolysis temperature and atmosphere in the process of heat treatment and provides the reference for the future experimental parameter design.

There are some creative methods for constructing high-performance MAMs. (i) Enhancing the shape anisotropy. Generally, the magnetic metal could maintain a high permeability in the frequency range of GHz if the Snoek’s limit is broke, and the introduction of shape anisotropy is considered as a promising method to realize this purpose [67, 173]. Enhancing shape anisotropy is beneficial for the improvement of natural resonance frequency and coercivity [174]. Therefore, the anisotropic magnetic materials such as thin film, flat plate and wire are worth trying to construct MAMs with high performance. For instant, Liu and co-workers synthesized porous Co/Ni/C nanosheets with CoNi-MOF-71 as precursors [76]. The Co/Ni/C nanosheets exhibited enhanced magnetic loss due to the great shape anisotropy of 2D Co/Ni, which provided higher coercivity and natural resonance frequency because of the break of Snoek’s limit. (ii) Widening aperture distribution. According to waveguide theory, the microwave could be dissipated inside the waveguide when the wavelength is twice the length of waveguide’s cross section [175]. Furthermore, different pore sizes correspond to different microwave length, which indicates that the gradient distribution of pore structure is beneficial to broaden the EAB [149]. Besides, macropores can facilitate the impedance match of MAMs, while mesopores and micropores can enhance the multiple reflection of microwave inside the MAMs [109]. There are many approaches to construct MAMs with multiple pore sizes such as etching [176] and template method [42]. (iii) Constructing Schottky contact structure. When the metals contact with the semiconductors, the semiconductor band at the interfaces will be bent to construct Schottky barrier, forming a significant region of space charge. The presence of Schottky barrier could hinder the smooth electron transfer, resulting in the accumulation of electrons at the interface and the enhancement of interface polarization [174]. Gao et al. prepared unique Fe-ZnO Schottky contact polycrystals by ligand exchange strategy and carbonization method [174]. The results shown that appropriate Schottky junction can improve impedance mismatch and enhance microwave loss of MAMs. (iv) Introducing sulfur in the pyrolysis process. Compared with metal oxides, corresponding metal sulfides have a greater variety of valence states and structures, as well as better electrical conductivity [177]. The sulfur powder sublimates at 450 °C, and the sulfur vapor can react with the metal atoms in the material to form sulfides with different valence states. Wu’ group prepared C/Cu/Cu_2_O@Cu_2-x_S and C/Cu_2−x_S/CuS absorbers using Cu-MOF-74 as precursors, indicating that the introduction of sulfide sulfur increased the number of dipoles and improve the conduction loss [178].

Although some achievements have been realized in the research and development of MOF-derived MAMs, there are still many significant challenges at this stage. First, the influence of various synthetic parameters on the synthesis of MOF-derived MAMs needs to be further explored. During preparation of MOFs, the reaction temperature and dosage ratio of raw materials, all have great effects on the sizes and morphologies of MOF precursors, which are directly related to the MA performance. Second, the microwave absorption mechanism needs to be further explored deeply. Microwave absorption capacity comes from the synergy of multi-mechanisms including dielectric loss, magnetic loss, multiple reflection and impedance matching, while the quantitative estimates on the influence of each mechanism still lack in sufficient theoretical supports. Besides, more well-designed MOF composites need to be made an attempt and deeply explored. The synergistic effect of multiple components is beneficial to the connectivity and energy conversion of electrical/magnetic networks in materials; MAMs derived from MOF composites are expected to bring new breakthroughs in this direction. Furthermore, MAMs with various excellent properties are waiting to be developed. In addition to strong absorption and wide band, the recently demand for MAMs is gradually developing toward the direction of high stability, multi-function and low cost to meet the requirements of actual application scenarios. Last, there is still a long way from research and development to industrial production and practical application. Current studies of MOF-derived MAMs only exist in the laboratory stage, and the mass production has not yet been achieved. In a word, the research on MOF-derived MAMs is still in the primary stage. The researchers still need to further clarify the MA mechanism and enrich the properties gradually based on a large number of experiments. In the meantime, researchers can scale up the experiment on the basis of the laboratory scale test and modify the process parameters during the pilot scale test, preparing for industrial production.

In conclusion, MOF-derived MAMs inject new blood into the field of microwave absorption due to their special structure, adjustable components and good electromagnetic properties. However, the research of MOF-derived MAMs is still in the infancy and there is much room for further research on the novel MOF-derived MAMs and their MA performance, in which the problems of practical application need to be focused on. We hope this comprehensive review can provide guidelines for the subsequent generation of MOF-derived MAMs and firmly believe that the challenges and issues will be solved unceasingly and MOF-derived MAMs has prodigious potential to achieve new breakthroughs in microwave absorption.
